# An Empirical Strategy for Characterizing Bacterial Proteomes across Species in the Absence of Genomic Sequences

**DOI:** 10.1371/journal.pone.0013968

**Published:** 2010-11-12

**Authors:** Joshua E. Turse, Matthew J. Marshall, James K. Fredrickson, Mary S. Lipton, Stephen J. Callister

**Affiliations:** Biological Sciences and Computational Sciences and Mathematics Division, Pacific Northwest National Laboratory, Richland, Washington, United States of America; Cairo University, Egypt

## Abstract

Global protein identification through current proteomics methods typically depends on the availability of sequenced genomes. In spite of increasingly high throughput sequencing technologies, this information is not available for every microorganism and rarely available for entire microbial communities. Nevertheless, the protein-level homology that exists between related bacteria makes it possible to extract biological information from the proteome of an organism or microbial community by using the genomic sequences of a near neighbor organism. Here, we demonstrate a trans-organism search strategy for determining the extent to which near-neighbor genome sequences can be applied to identify proteins in unsequenced environmental isolates. In proof of concept testing, we found that within a CLUSTAL W distance of 0.089, near-neighbor genomes successfully identified a high percentage of proteins within an organism. Application of this strategy to characterize environmental bacterial isolates lacking sequenced genomes, but having 16S rDNA sequence similarity to *Shewanella* resulted in the identification of 300–500 proteins in each strain. The majority of identified pathways mapped to core processes, as well as to processes unique to the *Shewanellae*, in particular to the presence of *c*-type cytochromes. Examples of core functional categories include energy metabolism, protein and nucleotide synthesis and cofactor biosynthesis, allowing classification of bacteria by observation of conserved processes. Additionally, within these core functionalities, we observed proteins involved in the alternative lactate utilization pathway, recently described in *Shewanella*.

## Introduction

Protein identification from peptide centric liquid chromatography-tandem mass spectrometry (LC-MS/MS)-based proteomics is currently limited to those organisms for which a genome or metagenome sequence is available. In the absence of sequence information, methods for identifying peptides include the use of *de novo* computational tools, as well as the use of trans-species comparisons or near neighbor genome sequences [1], [2]. Although interpretation of mass spectra using *de novo* tools has made considerable progress, the approach remains challenged by the shear number of possible amino acid sequence interpretations for measured fragmentation mass spectrum [3], [4]. Additionally, within any automated LC-MS/MS proteomics run, a large number of common contaminants are present [5]. Typically, masses derived from peptides belonging to these background proteins do not affect conventional searches. However, many of the proteins associated with contaminants, such as the keratins, contain large stretches of low complexity searches, which hit many other unrelated proteins in a sequence database search. Deconvolution and assignation of these low complexity regions to a single protein is difficult, if not impossible.

Recently, the UStags *de novo* approach [6] was published. As with other sequence tag identification strategies, UStags makes the assumption that ambiguous amino acids are located near the N- or C- terminus of a protein, regions that are usually more conserved[7], [8], [9], [10], [11]. Stretches of amino acids as small as 4 residues can be unique, allowing identification of a protein, using a peptide with ambiguous amino acids. However, as an error tolerant search, resulting candidate lists are large and require manual curation, though development of statistical models and automated filtering methodologies is underway [12], [13], [14].

An alternate approach involves using the genome from one organism to investigate the proteome of an unsequenced organism, which has been computationally investigated and experimentally demonstrated [1], [4], [12], [15], [16]. However, this approach has been constrained to bivariate comparisons and to comparisons within different strains of the same species. The majority of these investigations employed the “MS BLAST” homology searching protocol developed by Shevchenko, et al. [2]. MS BLAST is a sequence-based search strategy that involves *de novo* peptide sequencing, followed by a BLAST search to identify candidate proteins from these sequences. However, none of these studies addressed the question of how closely related an organism needs to be to generate meaningful data, especially, when multiple near neighbor (multiple species, strains, etc.) genome sequences exist.

In this study, we employed a systematic peptide identification strategy in which spectra derived from one organism were searched against the genome sequences of progressively more genetically distant neighbor organisms to measure the extent to which proteomic information could be obtained about one species when using the genomic sequence of another. Multiple genome sequences for *Shewanellae* were selected for proof of concept, not only because of the large number of publicly available genome sequences, but also because of the potential environmental importance of these organisms [17], [18], [19], [20], [21]. We also included sequences from two bacteria that are relatively distant from *Shewanellae*, i.e., *Deinococcus radiodurans* R1 and *Salmonella enterica* subsp. *enterica* serotype Typhimurium LT2 (*S.* Typhimurium) [22], [23], [24]. In an initial demonstration, we applied the strategy to identify proteins in four environment isolates of *Shewanella* obtained from sediments along the Columbia River in Washington state that lacked sequenced genomes [25]. These isolates had been identified as *Shewanella* by partial 16S rDNA sequencing. Depending upon the isolate, we identified 300–500 proteins from ∼4300 open reading frames based on sequenced *Shewanellae*. Note that species and strain designations are as in [26], except *Shewanella putrefaciens* CN32, which was originally described in [27].

Similar to most high throughput, mass spectrometry driven proteomic experiments, millions of unique spectra were generated for this empirical study, then analyzed using software tools that match measured spectra to a database of *in silico* spectra derived from genomic information. Ultimately, these tools allow for the identification of peptides and their parent proteins. Application of these tools to organisms without genome sequences (the approach demonstrated in this empirical study) is relatively new. In the future, emerging technologies, using a combination of de novo sequencing or unique sequence tags (UStags) may help expand the number of identified proteins, allowing further exploration of uncharacterized organisms.

## Results and Discussion

### Proof of concept

#### Global proteomics analysis

Spectra derived from previous studies of 11 *Shewanella* species, *D. radiodurans*, and *S.* Typhimurium were searched against their own genome sequences using the open source software tool X!Tandem [28], [29]. A total of 2,502,088 unique and fully tryptic peptide sequences containing at least six amino acid residues were identified and then filtered according to an X!Tandem calculated E-value of ≤5.01×10^−09^ to generate a list of the top 10% identified peptides. From these peptides, 30,528 proteins were identified by at least two unique peptides, and 26,539 of these proteins were observed in at least two organisms. The high degree of expressed protein homologs among the *Shewanella* organisms was expected because all were cultured aerobically in tryptic soy broth at 30°C. Tryptic soy broth represents a “universal medium” without going through an extended optimization process to develop a defined medium. Given the range of habitats the environmental isolates came from, tryptic soy broth was used minimize growth medium-related effects. The number of peptides/proteins identified for each organism was assumed to represent the maximum observable proteome for the particular growth and LC-MS/MS instrument conditions employed in this study.

#### Relationship between proteome and evolutionary distance of neighbor organisms

**Figure 1:**
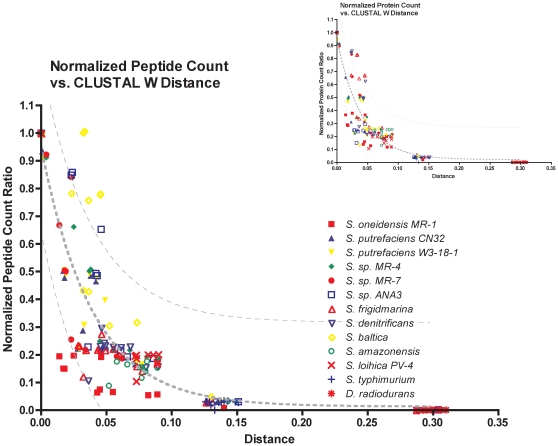
Peptide conservation (inset: protein conservation) was examined across the different species by graphing the normalized number of observed peptides (proteins) with respect to evolutionary distance. As the distance increases, the number of successfully identified features decreases. Data were fit to a one-phase exponential decay; 95% confidence interval for the observed features is shown with a hashed line. Each point represents the reference proteome peptide count relative to the near neighbor peptide count.

Spectra derived from a single condition for each organism were searched against the genome sequences of progressively more genetically distant (based on 16S-rDNA sequences) neighboring organisms. Normalized peptide/protein observation ratios were calculated by dividing the number of peptides/proteins identified (not observation count) for a particular organism when using the neighbor genome sequence into the number of peptides/proteins identified when using its own genome sequence. For example, spectra obtained for *Shewanella* sp. MR-7 that were searched against the *Shewanella* sp. MR-7 genome sequence yielded 4594 peptides. A search of the same spectra against the genome of near neighbor *Shewanella oneidensis* MR-1 yielded 3067 peptide identifications for a normalized peptide observation ratio of 0.67 (3067/4594). The normalized peptide ratios were plotted against evolutionary distances determined by CLUSTAL W [30], [31] (Table S1) and 16S rDNA (Figure 1) to examine the extent to which the genomic sequence of one organism can be used to identify proteins in another. Plots of the number of peptide (Figure S1) and protein (Figure S2) observations prior to normalization versus neighbor organism evolutionary distance also were generated for comparison.

Figure 1 shows that the numbers of observed peptides decrease as the evolutionary distance between an organism and its neighbors increases. The most rapid decrease appears between evolutionary distances of 0 to 0.05. This trend also is conserved across all organisms at the protein level (Figure 1 inset). Note that *S. putrefaciens* CN32 appears most closely related to *S. oneidensis* MR-1 (evolutionary distance of 0.016) and shares 4394 peptides observed in common. At approximate mid evolutionary distance (0.038), *S. frigidmarina* NCIMB400 shares only 1302 peptides with *S. oneidensis*. Between the two most genetically distant *Shewanellae*, i.e., *S. oneidensis* MR-1 and *S. amazonensis* SB2B (relative evolutionary distance 0.089), the number of peptides observed in common is 575, which means only 6% of the *S. oneidensis* MR-1 peptides are identified when searching *S. oneidensis* MR-1 spectra against the neighbor *S. amazonensis* SB2B genome. Furthermore, only 94 (0.9%) *Shewanella* peptides are identified when the *S*. Typhimurium LT2 (considered an outlier at an evolutionary distance of 0.11) genome is used to search the *Shewanella* spectra. Doubling the evolutionary distance to 0.299 (*D. radiodurans* R1) further decreases the number of identifications to a single peptide, i.e., insufficient peptide sequences for protein identification at these evolutionary distances (Figure 1 inset).

#### Comparison of protein functions assigned to observed orthologs

**Figure 2:**
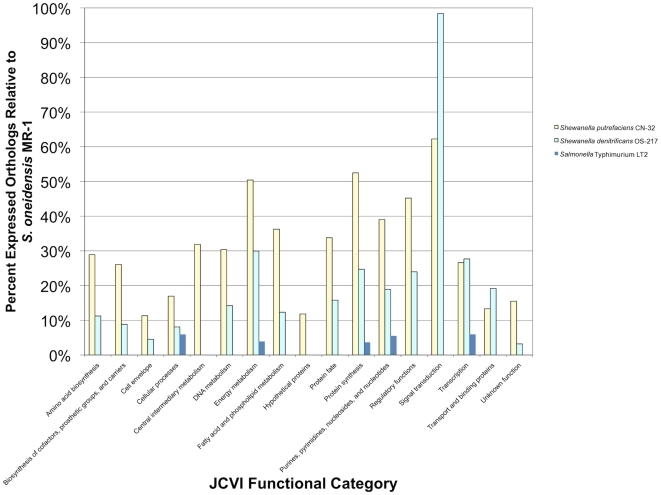
Conservation of functional orthologs across four of the species in the study is displayed, using normalized protein observations. Normalized protein observations were derived by dividing observed proteins for a single species within a category by the total number of proteins observed in the study. *S. oneidensis* orthologous groups from the JCVI Comprehensive Microbial Resource were employed to examine conservation of function.

Using the proteins identified from searching the *S. oneidensis* MR-1 spectra against the genomes of *S. putrefaciens* CN32, *S. denitrificans* OS217, and *S.* Typhimurium LT2, orthologs were mapped to functional categories to determine the level of conservation of protein function among the organisms. The latter three organisms represent near, mid, and remote evolutionary distances relative to *S. oneidensis* MR-1. Figure 2 attests to the genetic similarity between *S. oneidensis* MR-1 and *S. putrefaciens* CN32 relative to the similarity between *S. oneidensis* MR-1 and the other two organisms. Note that 50% of orthologs within energy metabolism and protein synthesis functional categories were observed when *S. oneidensis* MR-1 spectra were searched against the *S. putrefaciens* CN32 genome sequence. After searching *S. oneidensis* MR-1 spectra against the mid distant neighbor *S. denitrificans* OS217 genome sequence, only 30% of orthologs were observed in the energy metabolism category and only 25% were observed in the protein synthesis category. When the *S.* Typhimurium LT2 genome sequence was used to identify peptides from *S. oneidensis* MR-1, only 15% of the total orthologs (not within a specific JCVI functional category) were observed. This low percentage of observed orthologs is due to the lack of genomic or proteomic sequence homology between the two organisms and highlights the fact that for a surrogate genome to be used for peptide/protein identification, the two organisms must be phylogentically close. For instance, MS/MS spectra may have been generated for a peptide in *S. oneidensis* that comes from an ortholog between *S. oneidensis* and *S*. Typhimurium, yet a lack of sequence conservation for this peptide explains why that MS/MS spectra was not conserved between the two organisms. Similarly, a high percentage of observed orthologs may occur between organisms with few predicted orthologs. For instance, between *S. denitrificans* and *S. oneidensis*, 31 predicted orthologous proteins fall within the signal transduction category, whereas *S. putrefaciens* has 49 predicted *S. oneidensis* orthologs. Because of the lower number of predicted orthologs between *S. oneidensis* and *S. denitrificans*, within this functional category the observed result appears somewhat anomalous.

### Application to environmental Shewanella isolates

Following proof of concept, we applied the empirical strategy for characterizing bacterial proteomes across species in the absence of genomic sequences to identify peptides and proteins in four environmental *Shewanella* isolates from the Hanford Reach region of the Columbia River in Washington state. Although these isolates lacked sequenced genomes, two have 16S ribosomal DNA sequences indicative of phylogenetic affiliation with *S. oneidensis* MR-1, and two others have 16S ribosomal sequences indicative of an affiliation with *S. putrefaciens* CN32 [27] (Table S1). LC-MS/MS spectra were obtained for the four isolates, which were then systematically searched against the genome sequence of each *Shewanella* to identify proteins. The four isolates (HRCR-1, -2, -4 and -5) were cultured under the same conditions used in previous studies performed with sequenced *Shewanella* to allow for comparison of proteomes.

#### Extent of proteome information available for the isolates

**Figure 3:**
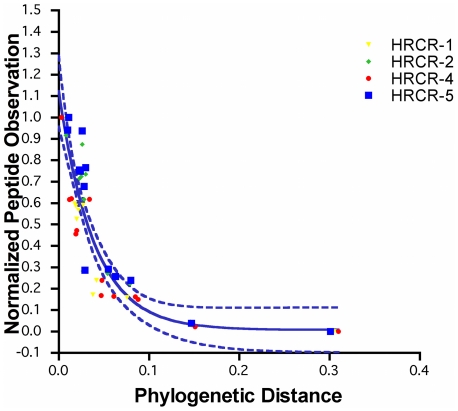
The number of peptides observed from the Columbia River *Shewanella* isolates graphed against evolutionary distance. The resulting trend agrees with the trend observed from characterized *Shewanella* species.

The number of peptides identified from each isolate was normalized to the number of near neighbor peptide identifications for each *Shewanella* and plotted against the neighbor evolutionary distance (Figure 3). Note that the resulting normalized data exhibit a sigmoidal regression line similar to the trans-organism comparison performed using *Shewanellae* with sequenced genomes, and peptide data points fall within the 95% prediction index. These results suggest that for these unsequenced *Shewanella* isolates, the sigmoidal regression curve can be used to predict the extent to which proteome information can be obtained from a sequenced near neighbor organism.

**Figure 4:**
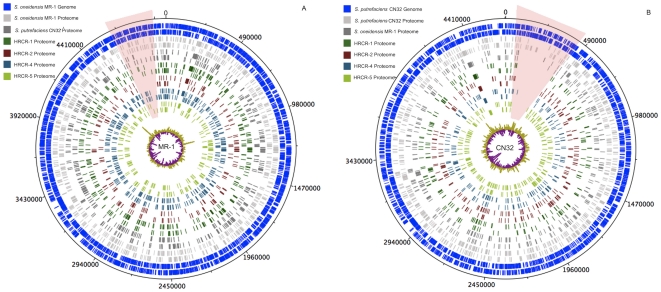
Protein identifications from the Columbia River isolates are mapped to the reference genomes *S. oneidensis* MR-1 (panel A) and *S. putrefaciens* CN32 (B). While all organisms were grown under the same conditions, observation of no protein expression compared to the reference proteome reveals that these organisms have undergone evolutionary divergence, which is reflected in protein expression. Also shown are the protein identifications for each of the *Shewanella* species mapped onto their respective genomes, as well as the protein orthologs across species. Two regions of ‘missing’ proteome information from the Hanford Reach isolates are highlighted.

The greatest number of proteins for the environmental isolates was observed when the genome sequences of either *S. oneidensis* MR-1 or *S. putrefaciens* CN32, i.e., the nearest phylogenetic neighbors of the isolates were utilized for protein identification. The extent of proteome similarity was revealed when proteins from the isolates were mapped to the genomes of *S. oneidensis* MR-1 and *S. putrefaciens* CN32 (Figure 4). Isolates HRCR-1 (457 proteins) and HRCR-4 (534 proteins) were observed most similar to the proteome of *S. oneidensis*, whereas the proteomes of HRCR-2 (276 proteins) and HRCR-5 (301 proteins) most similar to the proteome of *S. putrefaciens* (Table 1).

**Table 1:** Conservation of peptides amongst *Shewanella* isolates from the Hanford Reach of the Columbia River.

	Closest Relative			Furthest Relative		
Isolate	Strain	CLUSTAL distance	Conserved Peptides/Proteins	Strain	CLUSTAL distance	Conserved Peptides/Proteins
HRCR-1	*S. oneidensis* MR-1	0.009	2302/457	*S. loihica* PV-4	0.087	344/72
HRCR-2	*S. putrefaciens* CN32	0.008	1268/276	*S. loihica* PV-4	0.079	310/66
HRCR-4	*S. oneidensis* MR-1	0.003	2504/534	*S. loihica* PV-4	0.088	378/81
HRCR-5	*S. putrefaciens* CN32	0.010	1407/301	*S. loihica* PV-4	0.080	357/78

Distances were calculated from aligned partial 16s rDNA sequences using the CLUSTAL W algorithm. See ref. [30] & [31].

In Figure 4, the proteins mapped to the *S. oneidensis* MR-1 and *S. putrefaciens* CN32 genome sequences show distinct regions where proteins from the isolates were either lacking or not observed (Table S2 and Table S3). Figure 4A highlights a representative slice from the *S. oneidensis* genome in which no proteins were observed for HRCR 2, while Figure 4B shows that no proteins from any of the isolates were observed over a 30,000 base pair region (274 genes). In both maps, gene GC content and protein hydrophobicity (plotted in the center of Figure 4A and B) provided no insight into why these proteins were not observed.

Within the shaded region of the map in Figure 4A (proteins mapped to the *S. oneidensis* MR-1 genome) are genes that have predicted functions for formate metabolism, including formate dehydrogenase (locus tags SO4507–SO4515), as well as cytochrome *c* oxidase (SO4606–SO4609). *S. oneidensis* MR-1 contains two described determinants encoding metal efflux proteins, i.e., the Czc heavy metal and the Cus copper/silver efflux families. Although within the general region of the genome, metal efflux proteins were not observed in any of the isolates. Previous studies have demonstrated tight regulatory control of copper response elements in both *Shewanella* and other Gram-negative bacteria[32], [33]. Proteins responding to copper stress are only observed under stress-inducing growth conditions. Members of the Czc family of proteins are less well characterized, but also appear to be regulated as tightly as the Cus efflux protein family [34]. The shaded region in Figure 4B (proteins mapped to the *S. putrefaciens* CN32 genome) also contains several genes that encode proteins associated with formate metabolism and metal efflux protein families. Other proteins in this region are linked to fumarate metabolism and an additional two proteins contain putative 4Fe-4S ferredoxin iron-sulfur binding domains (locus tags CN32_0332, CN32_0336).

The absence of observed proteins in these regions could be due to ecoparalogy, where nucleotide substitutions in genes lead to differential regulation under the influence of a mutant regulator [35]. Ecoparalogy can result in an underestimation of the amount of protein information available when using a near neighbor organism genome sequence. Another plausible explanation for the absence of observed proteins in these regions may be linked to the growth of the organisms under study in highly aerated, rich growth medium. It is possible that a low nutrient, defined minimal medium may be more representative of the environment (i.e., Columbia River water/sediments) from which these bacteria were isolated. Growth of the Columbia River isolates under different nutritional conditions may result in a different complement of proteins expressed by the isolates, allowing investigation of alternate pathways, regulation, and protein expression within these regions. Alternatively, the lack of proteins in this region may simply be due to the absence of genes encoding these proteins in the isolate strains.

#### Proteome characterization of the isolates

*Shewanellae* are capable of using a vast respiratory network to reduce various organic and non-organic electron acceptors[1]. The utilization of a wide array of electron acceptors can be attributed to a large number of *c*-type cytochromes [1], [36], which have been shown to function as terminal reductases of metals [37], [38], [39] and radionuclides [40]. Within *S. oneidensis* MR-1 there are 42 putative *c*-type cytochromes that are expressed under a variety of conditions [41]. Under the nutrient rich, aerobic growth conditions used for this experiment, nine of the predicted *c*-type cytochromes were observed from the *S. oneidensis* MR-1 cultures (SO0970, SO1127, SO1778, SO1779, SO2178, SO2361, SO2363, SO2785, SO3420, SO4048, SO4666), while only two were detected in the *S. putrefaciens* CN32 cultures (CN32_0905, CN32_1958) (Table 2). The tetraheme cytochrome, fumarate reductase (SO0970 and CN32_0905) was observed in all isolates, suggesting that these isolates should be capable of fumarate respiration [42].

**Table 2:** *Shewanella* isolates were identified from the Columbia River, based on 16S rDNA sequencing.

		Number of identified peptides								
		*S. oneidensis* genome comparison					*S. putrefaciens* genome comparison			
MR-1 locus	CN32 locus	HRCR-1	HRCR-2	HRCR-4	HRCR-5		HRCR-1	HRCR-2	HRCR-4	HRCR-5
SO0970	CN32_0905	13	8	11	8		7	12	6	9
SO1777	CN32_1477									
SO1778	CN32_1478	11	3	8	4					
SO1779	CN32_1479			5						
SO2178	CN32_2287			2						2
SO2361	CN32_1956	6		4			2	2		3
SO2363	CN32_1958	3		5			3		5	
SO3420	CN32_2738	4	3	4	3					
SO3980	CN32_0685	3	2	2						
SO4666	CN32_3908			6						

Peptides from these isolates were compared to 11 sequenced *Shewanella* genomes, with the result that the most peptides were identified when *S. oneidensis* MR-1 or *S. putrefaciens* CN32 was used as the reference genome. Data below shows the number of identified peptides from the *c*-type cytochromes using either *S. oneidensis* MR -1 or *S. putrefaciens* CN32 genomes.

Two other cytochromes (SO1778 and SO3420) were identified in all isolates when the *S. oneidensis* genome was employed for protein identification (Table 2). SO1778 is a decaheme cytochrome *c*, MtrC (OmcB) that has been implicated in metal and radionuclide reduction by *S. oneidensis* MR-1 [43], [44], [45], [46], [47]. In both *S. oneidensis* MR-1 and *S. putrefaciens* CN32, *omcB* is part of a metal reductase-containting locus that is typically co-expressed with *omcA* (SO1779), *mtrA* (SO1777) and *mtrB* (SO1776) [1], [36], so it is surprising that an OmcA homolog was observed in just one of the isolates, i.e., HRCR-4 (Table 2). A plausible explanation is that these cytochromes were not observed because of the high variability of mass spectrometry based proteomics. The second cytochrome detected, SO3420 is a cytochrome *c'* with little functional characterization and previously predicted to be a cytochrome solely through comparative genomic studies [48], [49].

*Shewanellae*'s promiscuity for terminal electron acceptors is matched by a variety of pathways available for assimilating carbon beyond central metabolism [50]. For example, lactate is a common carbon and energy substrate for *Shewanella* that is oxidized completely under aerobic conditions and oxidized incompletely to acetate under anaerobic growth conditions. Similar to 2-oxoglutarate, the enzyme lactate dehydrogenase (*dld;* SO0968) was only observed in HRCR-1 and HRCR-4 when the *S. oneidensis* MR-1 genome sequence was used to identify proteins in the isolates. When the *S. putrefaciens* CN32 genome sequence was used, only the lactate dehydrogenase in HRCR-5 was observed. Pinchuk, et al. demonstrated the presence of an alternative lactate utilization pathway in *S. oneidensis* MR-1 [51], and we observed protein components (LldF, SO1519 and Lld-II, SO1521) of this second pathway in all isolates. While orthologs featuring similar topology for this second lactate utilization pathway exist in *S. putrefaciens* CN32, we only observed these orthologs in HRCR-1, -2, and -5, with HRCR-1 exhibiting two of the three proteins from L-lactate dehydrogenase and the entire D-lactate dehydrogenase. Differential observation of the components of these two lactate pathways across the proteomes is likely due to sequence divergence between *S. oneidensis* MR-1 and *S. putrefaciens* CN32.

Characterization of proteins associated with the glycolytic and TCA metabolic pathways in the isolates revealed little difference in the number of observed proteins within these pathways, regardless of the *Shewanella* genome sequence used for identification (Table 3). For example, with the exception of a few proteins, representation of glycolysis and the TCA cycle was complete, which implies that the proteins making up these pathways are part of the core proteome [52] associated with *Shewanella*. The exception encompassed four proteins in the glycolytic pathways (SO2486–SO2489 or CN32_1866- CN32_1869) involved in the conversion of glucose-6-phosphate to glyceraldehyde-3-phosphate (the pentose phosphate pathway). Across all of the environmental *Shewanella* isolates, only one enzyme in the pentose phosphate pathway, phosphogluconate dehydratase (Edd, SO2487 and CN32_1868) was observed. When the *S. oneidensis* MR-1 genome sequence was used to identify proteins expressed by the isolates, phosphogluconate dehydratase was observed in those strains that were more closely related to *S. oneidensis* MR-1, i.e., HRCR-1 and HRCR-4. This pattern was retained when the *S. putrefaciens* CN32 genome sequence was used for protein identification, i.e., phosphogluconate dehydratase was only observed in HRCR-2 and HRCR-5, which are the two strains most similar to *S. putrefaciens* CN32 (Table 4).

**Table 3:** *S. oneidensis* MR-1 peptide fragmentation patterns where mapped to theoretical spectra from organisms representing near, mid, and distant phylogenetic neighbors.

		Unique Peptides Identified			
Locus	Gene	*S. oneidensis* MR-1	*S. putrefaciens* CN32	*S. denitrificans* OS217	*S.* Typhimurium
Glycolysis/Entner-Doudoroff Pathway					
SO0049	*gpmA*	5			
SO0932	*pgk*	8	2		
SO0933	*fba*	10	4	4	
SO1200	*tpiA*	3			
SO2345	*gapA-2*	7	3		
SO2347	*gapA-3*	4			
SO2486	*eda*	3	2		
SO2487	*edd*	5			
SO2488	*pgl*	2			
SO2489	*zwf*	4			
SO2491	*pykA*	10	4		
SO2644	*ppsA*	15	6	3	
SO3440	*eno*	8	3	2	2
SO3547	*pgi*	3			
SO3991	*fbp*	4	3	2	
TCA Cycle					
SO0343	*acnA*	5	3		
SO0344	*prpC*	4			
SO0432	*acnB*	30	10	3	
SO0770	*mdh*	10	7	5	2
SO0970	*SO0970*	11	4		
SO1484	*aceA*	2			
SO1926	*gltA*	6	4	2	
SO1928	*sdhA*	5	4	2	
SO1929	*sdhB*				
SO1930	*sucA*				
SO1931	*sucB*	9			
SO1932	*sucC*	5	2		
SO1933	*sucD*	6	4		
SO2222	*SO2222*	12	2		
SO2629	*icd*	50	12	4	
SO4118	*SO4118*	4			

Data represented here are from highly conserved central metabolic pathways, as unique peptide count – the number of peptides identified belonging to a protein with an ortholog in the *S. oneidensis* genome.

**Table 4:** *Shewanella* isolates were identified from the Columbia River, based on 16S rDNA sequencing.

			Number of identified peptides								
			*S. oneidensis* genome comparison					*S. putrefaciens* genome comparison			
MR-1 locus	CN32 locus	Gene	HRCR-1	HRCR-2	HRCR-4	HRCR-5		HRCR-1	HRCR-2	HRCR-4	HRCR-5
Glycolysis/Entner-Doudoroff Pathway											
SO0049	CN32_0040	*gpm*A			3			2			
SO0932	CN32_0874	*pgk*	7	5	7	5		5	3	4	4
SO0933	CN32_0875	*fba*	6	6	5	6		4	5	4	5
SO1200	CN32_2838	*tpi*A		3	3	4		3		3	3
SO2345	CN32_1889	*gap*A-2	4	3	2	3		3	3	2	2
SO2347	CN32_1891	*gap*A-3	6	3	5	3		3	2	3	4
SO2486	CN32_1869	*eda*									
SO2487	CN32_1868	*edd*	2		2				2		3
SO2488	CN32_1867	*pgl*									
SO2489	CN32_1866	*zwf*									
SO2491	CN32_1864	*pyk*A	3	8	4	6			5	3	5
SO2644	CN32_2243	*pps*A	11	7	9	8		8	4	5	3
SO3440	CN32_2757	*eno*	6	7	6	7		3	6	2	5
SO3547	CN32_1048	*pgi*	2		2						
SO3991	CN32_0676	*fbp*	5	3	5	4		4	3	3	4
TCA Cycle											
SO0343	CN32_3646	*acn*A	4	4	5	3		4	4	4	5
SO0344	CN32_3645	*prp*C	2	2	3	3		2	3	2	3
SO0432	CN32_3409	*acn*B	19	13	19	12		14	12	11	12
SO0770	CN32_3219	*mdh*	6	5	7	5		5	4	6	3
SO0970	CN32_0905	SO0970	13	8	11	8		7	12	6	9
SO1484	CN32_1239	*ace*A		3	2	2			2	2	2
SO1926	CN32_2274	*glt*A	4	4	5	6		5	3	3	4
SO1928	CN32_2271	*sdh*A	7	5	3	3		5	5	2	2
SO1929	CN32_2270	*sdh*B									
SO1930	CN32_2269	*suc*A	6	4	9	4		2	4	4	4
SO1931	CN32_2268	*suc*B	4	4	6	4		4	3	3	4
SO1932	CN32_2267	*suc*C	3	2	4	3		2	2	2	
SO1933	CN32_2266	*suc*D	4	2	4	2		4	2	4	
SO2222	CN32_1807	SO2222	7	6	5	5		5	8	6	6
SO2629	CN32_2230	*icd*	15	5	12	10		8	7	6	11
SO4118	CN32_0554	SO4118	2		2						

Peptides from these isolates were compared to 11 sequenced *Shewanella* genomes, with the result that the most peptides were identified when *S. oneidensis* MR-1 or *S. putrefaciens* CN32 was used as the reference genome. Data below shows the number of identified peptides from central metabolic pathways using either *S. oneidensis* MR -1 or *S. putrefaciens* CN32 genomes.

A high percentage of the TCA cycle proteins were observed in all isolates (Table 3). For example, 2-oxoglutarate dehydrogenase, a member of a three-enzyme complex that converts alpha-glutarate to succinyl-CoA was observed in each of the isolates, but not observed in the proteomes of either *S. oneidensis* MR-1 or *S. putrefaciens* CN32. Observation of this protein in the isolates and the concomitant lack of observation in *S. oneidensis* MR-1 and *S. putrefaciens* CN32 may be due to a difference in growth stage or regulatory control, causing 2-oxoglutarate dehydrogenase to be present in greater abundance in the environmental *Shewanella* isolates.

We demonstrated a strategy for selecting and utilizing near neighbor organism genome sequences that enabled proteomics characterization of unsequenced environmental isolates lacking sequenced genomes. In spite of the fact that rapid microbial bacterial genome sequencing is becoming increasingly affordable, it is not yet practical to generate whole genome sequences for all organisms isolated from a complex environmental sample nor may it be warranted.

The proof of concept portion of this study revealed that the largest number of peptide identifications for an organism resulted when the evolutionary distance of the sequenced neighbor fell within 0–0.046, after which the extent of proteome characterization derived from a near neighbor genome decreased as evolutionary distance increased. Application of the strategy to characterize Columbia River *Shewanella* isolates revealed that the *Shewanella* were genetically related to either *S. oneidensis* MR-1 or *Shewanella putrefaciens* CN32. In the absence of whole genome sequences for these isolates, application of the strategy also resulted in the identification of 300–500 proteins, which represents the first proteome characterization of these isolates beyond partial 16S rDNA sequencing. As demonstrated here, there is a limit to how close a near-neighbor genome needs to be in order to make meaningful protein identification, within confidence limits. However, the proteome information generated provided a starting point for elucidating underlying metabolic networks that define adaptation to different environments and ultimately speciation [53], [54], [55]. Tandem mass spectrometry data for the isolates is available through the Biological MS Data and Software Distribution Center website at http://omics.pnl.gov.

With the careful application of error-tolerant search methodologies, such as *de novo* peptide sequencing, or the USTags approach [8], additional identifications of orthologous proteins that contain sequence polymorphisms may result. Additionally, the generation of high-resolution tandem mass spectra may improve quality and confidence scores associated with spectral matching and *de novo* tools, resulting in a larger number of proteins identified (see citations [56], [57]for reviews).

## Materials and Methods

### Bacterial growth conditions

In earlier studies, *Shewanella* sp. samples analyzed using LC-MS/MS to generate peptide reference databases for the *Shewanella* Federation were grown aerobically in tryptic soy broth without dextrose (BD Diagnostics, Sparks, MD, USA) at 30°C with shaking at 200 rpm to an OD_600_ ∼0.5. In other earlier studies, *Salmonella* serovar Typhimurium strain LT2 was grown in Luria-Bertani broth [58] at 37°C and *Deinococcus radiodurans* R1, in TGY medium at 30°C. Cells were harvested by centrifugation (8000× g for 10 min at 4°C), flash frozen in liquid nitrogen, and then stored at –80°C until processing. Environmental *Shewanella* isolates were obtained from samples of the water-sediment interface in the Hanford Reach region of the Columbia River near Richland, Washington [27].

Proteins were prepared as outlined in Lipton, et al. [59]. In brief, cells were lysed by bead beating in 100 mM NH_4_HCO_3_ buffer (pH ∼8). Proteins were eluted and denatured with 7M urea, 2M thiourea, and 5 mM DTT at 60°C for 30 min. For soluble and insoluble analyses, cell pellets were treated as above, and the lysate was centrifuged. The supernatant (soluble preparation) was transferred to a fresh tube, and the remaining pellet was resuspended in 7M urea, 2M thiourea, 1% CHAPS in 50 mM NH_4_HCO_3_, and 5 mM DTT at 60°C for 30 min (insoluble preparation). For all analyses, the denatured proteins were diluted with buffer to reduce the salt concentration and digested with trypsin for 3 h at 37°C. Cleanup was performed by passing the samples through a C18 SPE column [60]. The sample solutions were concentrated in a speed-vac to a final volume of ∼50–100 µL, quick frozen in liquid nitrogen, and stored at –80°C until needed for analysis.

Samples were fractionated by strong cation exchange chromatography [59]. Approximately 25 fractions were collected from each sample, and each fraction was dried under vacuum and then dissolved in 30 µL of 25 mM NH_4_HCO_3_. Aliquots containing 10 µg of protein were analyzed by LC-MS/MS, using an LTQ ion trap mass spectrometer (ThermoFisher Scientific Corp., San Jose, CA) and previously defined parameters [61].

### Peptide/protein identification using a trans-organism search strategy

The X!Tandem algorithm [28], [29] was employed to match MS/MS spectra with predicted tryptic peptides from a protein file. Our search strategy allowed for partial tryptic peptides to pass the first round of searching by X!Tandem. The scores produced by X!Tandem are probability-based scores similar to the E-value or bit score from BLAST. Genomic sequences for each bacterial species were obtained from publicly available databases.

Spectra for each of the bacterial samples were systematically searched relative to the translated genome sequences of all species to identify common peptides. *Salmonella* and *Deinococcus* were included as outliers, similar to the inclusion of distantly related organisms when constructing and calculating confidence of genetic trees [62], [63]. A total of 4261 X!Tandem searches were performed using the PRISM computing cluster (260 days of CPU time across 32 processing nodes) [64]. Percentages of observed orthologs were calculated as the number of orthologs observed from *S. oneidensis* MR-1 spectra when searched using one of the three neighboring genome sequences divided by the number of orthologs observed from the same *S. oneidensis* MR-1 spectra when searched against its own genome sequence.

### Data analysis of X!Tandem results

For the Shewanella species in this study, distribution of X!Tandem log-transformed E-values was divided into five intervals. Intervals represented the 10^th^ (Interval A; all values ≤−8.3), 25^th^ (−8.3> Interval B≤−6.1), 50^th^ (−6.1 > Interval C≤−3.7), 75^th^ (−3.7> Interval D ≤−1.6) and 90^th^ (Interval E; all values >−1.6) percentiles. Only fully tryptic peptides having a minimum amino acid length of 6 residues and a log E-value ≤−8.3 were used in this evaluation. A protein was considered positively observed when identified by at least two unique peptides.

Regression analysis was performed in GraphPad Prism (GraphPad Software, Inc., La Jolla, CA). Several nonlinear regression models were tested, including exponential decay and polynomial association. The simplest model with the largest R^2^ value and most significant F-test was selected as the model with the best fit (in this case a two sigmoidal dose-response model).

A 95% prediction index rather than 95% confidence interval was calculated using GraphPad Prism (GraphPad Software, Inc.). This index was used to predict the next Y value for a given X, which in this case was the number of peptides/proteins for a specified evolutionary distance from a neighboring strain or species. Unlike confidence intervals obtained for replicate data, a prediction interval was used in cases where there was only a single observation of Y. Because the uncertainty of each peptide identification was unknown, all observations were given the same weight.

### Near neighbor evolutionary distance calculation

Because of the small amount of sequence data available for each isolate, a partial 16S rDNA sequence that represented the 5′ end of the 16S rDNA gene (850 bp) was used to generate the CLUSTAL W genetic distance matrix. Sequence alignment was accomplished using the CLUSTAL W alignment algorithm accessed from the San Diego Supercomputing Center [65], [66]. Near neighbor evolutionary distances were reported as CLUSTAL W distances.

## Supporting Information

Table S1 Sequences for 16S rRNA were used for determination of evolutionary distance between Shewanella strains and the outlier species, Salmonella Typhimurium LT2 and Deinococcus radiodurans R1. Distance calculations were carried out using CLUSTAL, hosted at the San Diego Supercomputer Center Biology Workbench (http://workbench.sdsc.edu/). Values are CLUSTAL distances.
(0.07 MB DOC)

Table S2 S. oneidensis MR-1 loci with poor proteome coverage from analysis with the Columbia River Shewanella isolates. ND indicates Not Detected, P indicates Present.
(0.57 MB DOC)

Table S3 S. putrefaciens CN32 loci with poor proteome coverage from analysis with the Columbia River Shewanella isolates. ND indicates Not Detected, P indicates Present.
(0.47 MB DOC)

Figure S1 Plot of the number of peptide observations prior to normalization versus neighbor organism evolutionary distance.
(0.11 MB TIF)

Figure S2 Plot of the number of protein observations prior to normalization versus neighbor organism evolutionary distance.
(0.10 MB TIF)
