# Supplementary material for: An Empirical Strategy for Characterizing Bacterial Proteomes across Species in the Absence of Genomic Sequences
Source: PLoS One. 2010 Nov 12;5(11):e13968. doi: 10.1371/journal.pone.0013968 (PMC2980473; doi:10.1371/journal.pone.0013968)
Supplement: Table S1 — Sequences for 16S rRNA were used for determination of evolutionary distance between Shewanella strains and the outlier species, Salmonella Typhimurium LT2 and Deinococcus radiodurans R1. Distance calculations were carried out using CLUSTAL, hosted at the San Diego Supercomputer Center Biology Workbench (http://workbench.sdsc.edu/). Values are CLUSTAL distances. (0.07 MB DOC) [file pone.0013968.s001.doc]

|  | *S. oneidensis* MR-1 | *S.* sp. MR-7 | *S. putrefaciens* CN32 | *S.* sp. W3-18-1 | *S.* sp. MR-4 | *S. frigidimarina* NCIMB400 | *S. baltica* OS155 | *S.* sp. ANA-3 | *S. denitrificans* NCIMB400 | *S. loihica* PV-4 | *S. amazonensis* SB2B | *S.* Typhimurium LT2 | *D. radiodurans* R1 | HRCR1 | HRCR2 | HRCR4 | HRCR5 |
| --- | --- | --- | --- | --- | --- | --- | --- | --- | --- | --- | --- | --- | --- | --- | --- | --- | --- |
| *S. oneidensis* MR-1 | 0 | 0.014 | 0.017 | 0.018 | 0.025 | 0.043 | 0.045 | 0.046 | 0.055 | 0.082 | 0.089 | 0.140 | 0.299 | 0.009 | 0.026 | 0.003 | 0.026 |
| *S.* sp. MR-7 | 0.014 | 0 | 0.018 | 0.019 | 0.004 | 0.034 | 0.023 | 0.023 | 0.045 | 0.062 | 0.058 | 0.127 | 0.304 | 0.018 | 0.025 | 0.012 | 0.023 |
| *S. putrefaciens* CN32 | 0.017 | 0.018 | 0 | 0.001 | 0.038 | 0.028 | 0.032 | 0.042 | 0.048 | 0.072 | 0.076 | 0.136 | 0.295 | 0.020 | 0.008 | 0.019 | 0.010 |
| *S.* sp. W3-18-1 | 0.018 | 0.019 | 0.001 | 0 | 0.037 | 0.029 | 0.033 | 0.043 | 0.049 | 0.073 | 0.077 | 0.138 | 0.293 | 0.022 | 0.009 | 0.020 | 0.011 |
| *S.* sp. MR-4 | 0.025 | 0.004 | 0.038 | 0.037 | 0 | 0.055 | 0.045 | 0.024 | 0.068 | 0.090 | 0.081 | 0.149 | 0.310 | 0.018 | 0.030 | 0.014 | 0.030 |
| *S. frigidimarina* NCIMB400 | 0.043 | 0.034 | 0.028 | 0.029 | 0.055 | 0 | 0.032 | 0.049 | 0.046 | 0.073 | 0.077 | 0.126 | 0.287 | 0.038 | 0.027 | 0.047 | 0.029 |
| *S. baltica* OS155 | 0.045 | 0.023 | 0.032 | 0.033 | 0.045 | 0.032 | 0 | 0.036 | 0.036 | 0.073 | 0.052 | 0.130 | 0.293 | 0.042 | 0.028 | 0.048 | 0.028 |
| *S.* sp. ANA-3 | 0.046 | 0.023 | 0.042 | 0.043 | 0.024 | 0.049 | 0.036 | 0 | 0.061 | 0.086 | 0.066 | 0.151 | 0.304 | 0.027 | 0.023 | 0.034 | 0.024 |
| *S. denitrificans* OS217 | 0.055 | 0.045 | 0.048 | 0.049 | 0.068 | 0.046 | 0.036 | 0.061 | 0 | 0.089 | 0.080 | 0.133 | 0.287 | 0.062 | 0.054 | 0.061 | 0.055 |
| *S. loihica* PV-4 | 0.082 | 0.062 | 0.072 | 0.073 | 0.090 | 0.073 | 0.073 | 0.086 | 0.089 | 0 | 0.089 | 0.129 | 0.304 | 0.087 | 0.079 | 0.088 | 0.080 |
| *S. amazonensis* SB2B | 0.089 | 0.058 | 0.076 | 0.077 | 0.081 | 0.077 | 0.052 | 0.066 | 0.080 | 0.089 | 0 | 0.143 | 0.306 | 0.076 | 0.062 | 0.085 | 0.063 |
| *S.* Typhimurium LT2 | 0.140 | 0.127 | 0.136 | 0.138 | 0.149 | 0.126 | 0.130 | 0.151 | 0.133 | 0.129 | 0.143 | 0 | 0.292 | 0.147 | 0.146 | 0.151 | 0.147 |
| *D. radiodurans* R1 | 0.299 | 0.304 | 0.295 | 0.293 | 0.310 | 0.287 | 0.293 | 0.304 | 0.287 | 0.304 | 0.306 | 0.292 | 0 | 0.305 | 0.301 | 0.310 | 0.301 |
| HRCR1 | 0.009 | 0.018 | 0.020 | 0.022 | 0.018 | 0.038 | 0.042 | 0.027 | 0.062 | 0.087 | 0.076 | 0.147 | 0.305 | 0 | 0.018 | 0.009 | 0.017 |
| HRCR2 | 0.026 | 0.025 | 0.008 | 0.009 | 0.030 | 0.027 | 0.028 | 0.023 | 0.054 | 0.079 | 0.062 | 0.146 | 0.301 | 0.018 | 0 | 0.026 | 0.001 |
| HRCR4 | 0.003 | 0.012 | 0.019 | 0.020 | 0.014 | 0.047 | 0.048 | 0.034 | 0.061 | 0.088 | 0.085 | 0.151 | 0.31 | 0.009 | 0.026 | 0 | 0.024 |
| HRCR5 | 0.026 | 0.023 | 0.010 | 0.011 | 0.030 | 0.029 | 0.028 | 0.024 | 0.055 | 0.080 | 0.063 | 0.147 | 0.301 | 0.017 | 0.001 | 0.024 | 0 |
