# Supplementary material for: An Empirical Strategy for Characterizing Bacterial Proteomes across Species in the Absence of Genomic Sequences
Source: PLoS One. 2010 Nov 12;5(11):e13968. doi: 10.1371/journal.pone.0013968 (PMC2980473; doi:10.1371/journal.pone.0013968)
Supplement: Table S2 — S. oneidensis MR-1 loci with poor proteome coverage from analysis with the Columbia River Shewanella isolates. ND indicates Not Detected, P indicates Present. (0.57 MB DOC) [file pone.0013968.s002.doc]

| Locus | Genome Start | Description | HRCR1 | HRCR2 | HRCR4 | HRCR5 |
| --- | --- | --- | --- | --- | --- | --- |
| SO_4318 | 4503564 | toxin secretion ATP-binding protein | ND | ND | ND | P |
| SO_4319 | 4505665 | HlyD family secretion protein | ND | ND | ND | ND |
| SO_4320 | 4507146 | agglutination protein | P | P | ND | P |
| SO_4321 | 4508558 | OmpA family protein | P | ND | ND | P |
| SO_4322 | 4509182 | conserved hypothetical protein | ND | ND | ND | ND |
| SO_4323 | 4509898 | GGDEF domain protein | ND | ND | ND | ND |
| SO_4324 | 4512635 | GGDEF domain protein | ND | ND | ND | ND |
| SO_4325 | 4515491 | ATP-dependent DNA helicase Rep | ND | ND | ND | ND |
| SO_4326 | 4517790 | transcriptional regulator, TetR family | ND | ND | ND | ND |
| SO_4327 | 4518424 | HlyD family secretion domain protein | ND | ND | ND | ND |
| SO_4329 | 4522763 | conserved hypothetical protein | ND | ND | ND | ND |
| SO_4330 | 4523186 | conserved hypothetical protein | ND | ND | ND | ND |
| SO_4331 | 4523578 | hypothetical protein | ND | ND | ND | ND |
| SO_4332 | 4523931 | conserved hypothetical protein | ND | ND | ND | ND |
| SO_4333 | 4525333 | hypothetical protein | ND | ND | ND | ND |
| SO_4334 | 4526146 | inner membrane protein, putative | ND | ND | ND | ND |
| SO_4335 | 4527624 | phosphatidylglycerophosphatase B, putative | ND | ND | ND | ND |
| SO_4336 | 4528464 | hypothetical protein | ND | ND | ND | ND |
| SO_4337 | 4528777 | transcriptional regulator, AsnC family | ND | ND | ND | ND |
| SO_4338 | 4529341 | hypothetical protein | ND | ND | ND | ND |
| SO_4339 | 4529617 | sodium-dependent transporter, putative | ND | ND | ND | ND |
| SO_4340 | 4531068 | conserved hypothetical protein | P | ND | ND | P |
| SO_4341 | 4532618 | hypothetical protein | ND | ND | ND | ND |
| SO_4342 | 4534088 | hypothetical protein | ND | ND | ND | ND |
| SO_4343 | 4534383 | aminotransferase, class V | P | ND | ND | P |
| SO_4344 | 4535656 | threonine dehydratase | ND | ND | ND | ND |
| SO_4345 | 4537295 | dihydroxy-acid dehydratase | ND | P | ND | ND |
| SO_4346 | 4539233 | acetolactate synthase II, small subunit | ND | ND | ND | ND |
| SO_4347 | 4539490 | acetolactate synthase II, large subunit | ND | ND | ND | ND |
| SO_4348 | 4541319 | hypothetical protein | ND | ND | ND | ND |
| SO_4349 | 4541742 | ketol-acid reductoisomerase | ND | P | ND | ND |
| SO_4350 | 4543417 | transcriptional regulator ilvY | ND | ND | ND | ND |
| SO_4351 | 4544347 | CBS domain protein | ND | ND | ND | ND |
| SO_4352 | 4546040 | hypothetical protein | ND | ND | ND | ND |
| SO_4353 | 4546129 | hypothetical protein | ND | ND | ND | ND |
| SO_4354 | 4546634 | conserved hypothetical protein | ND | ND | ND | ND |
| SO_4355 | 4547271 | hypothetical protein | ND | ND | ND | ND |
| SO_4356 | 4548063 | conserved domain protein | ND | ND | ND | ND |
| SO_4357 | 4550380 | anaerobic dimethyl sulfoxide reductase, B subunit | ND | ND | ND | ND |
| SO_4358 | 4551016 | anaerobic dimethyl sulfoxide reductase, A subunit | ND | ND | ND | ND |
| SO_4359 | 4553623 | outer membrane protein, putative | ND | ND | ND | ND |
| SO_4360 | 4555605 | decaheme cytochrome c | ND | ND | ND | ND |
| SO_4361 | 4556587 | hypothetical protein | ND | ND | ND | ND |
| SO_4362 | 4557054 | conserved hypothetical protein | ND | ND | ND | ND |
| SO_4363 | 4558218 | hypothetical protein | ND | ND | ND | ND |
| SO_4364 | 4558251 | ATP-dependent DNA helicase RecG | ND | ND | ND | ND |
| SO_4365 | 4560496 | hypothetical protein | P | P | ND | P |
| SO_4366 | 4561760 | conserved hypothetical protein | ND | ND | ND | ND |
| SO_4367 | 4562493 | acyltransferase family protein | ND | ND | ND | ND |
| SO_4368 | 4563328 | acyl carrier protein, putative | ND | ND | ND | ND |
| SO_4369 | 4563592 | acyl carrier protein, putative | ND | ND | ND | ND |
| SO_4370 | 4563840 | membrane protein, putative | ND | ND | ND | ND |
| SO_4371 | 4564385 | conserved hypothetical protein | ND | ND | ND | ND |
| SO_4372 | 4565742 | thioester dehydrase family protein | ND | ND | ND | ND |
| SO_4373 | 4566149 | glycosyl transferase, group 2 family protein | ND | ND | ND | ND |
| SO_4374 | 4567930 | histidine ammonia-lyase, putative | ND | ND | ND | ND |
| SO_4375 | 4569492 | conserved hypothetical protein | ND | ND | ND | ND |
| SO_4376 | 4569923 | hypothetical protein | ND | ND | ND | ND |
| SO_4377 | 4570753 | membrane protein, putative | ND | ND | ND | ND |
| SO_4378 | 4573258 | FAD-binding protein | ND | ND | ND | ND |
| SO_4379 | 4574573 | hypothetical protein | ND | ND | ND | ND |
| SO_4380 | 4575430 | 3-oxoacyl-(acyl-carrier-protein) synthase II, putative | ND | ND | ND | ND |
| SO_4381 | 4576653 | thioester dehydrase family protein | ND | ND | ND | ND |
| SO_4382 | 4577300 | 3-oxoacyl-(acyl-carrier-protein) reductase | ND | ND | ND | ND |
| SO_4383 | 4578026 | 3-oxoacyl-(acyl-carrier-protein) synthase II | ND | ND | ND | ND |
| SO_4384 | 4579299 | hypothetical protein | P | ND | ND | ND |
| SO_4385 | 4580321 | von Willebrand factor type A domain protein | ND | ND | ND | ND |
| SO_4386 | 4581797 | ISSod4, transposase | ND | ND | ND | ND |
| SO_4387 | 4583032 | sensor histidine kinase | ND | ND | ND | ND |
| SO_4388 | 4584567 | DNA-binding response regulator | ND | ND | ND | ND |
| SO_4389 | 4585328 | hypothetical protein | ND | ND | ND | ND |
| SO_4391 | 4587674 | hypothetical protein | ND | ND | ND | ND |
| SO_4392 | 4588537 | hypothetical protein | ND | ND | ND | ND |
| SO_4393 | 4589077 | acetyltransferase, GNAT family | ND | ND | ND | ND |
| SO_4394 | 4589665 | phage shock protein E | ND | ND | ND | ND |
| SO_4395 | 4590031 | hypothetical protein | ND | ND | ND | ND |
| SO_4396 | 4590257 | acyl carrier protein phosphodiesterase | ND | ND | ND | ND |
| SO_4397 | 4590988 | conserved hypothetical protein | ND | ND | ND | ND |
| SO_4398 | 4591451 | conserved hypothetical protein TIGR00256 | ND | ND | ND | ND |
| SO_4399 | 4591904 | hypothetical protein | ND | ND | ND | ND |
| SO_4400 | 4592515 | proline iminopeptidase, putative | ND | ND | ND | ND |
| SO_4401 | 4593675 | ribonuclease BN | ND | ND | ND | ND |
| SO_4402 | 4594641 | hypothetical protein | ND | ND | ND | ND |
| SO_4403 | 4594880 | hypothetical protein | ND | ND | ND | ND |
| SO_4404 | 4595949 | iron-sulfur cluster-binding protein | ND | ND | ND | ND |
| SO_4405 | 4597190 | catalase/peroxidase HPI | ND | ND | ND | ND |
| SO_4406 | 4599438 | hypothetical protein | ND | ND | ND | ND |
| SO_4407 | 4599742 | GGDEF family protein | ND | ND | ND | ND |
| SO_4408 | 4600865 | virulence regulator BipA | P | P | ND | P |
| SO_4409 | 4602910 | hypothetical protein | ND | ND | ND | ND |
| SO_4410 | 4603654 | glutamine synthetase, type I | P | P | P | P |
| SO_4411 | 4605328 | hypothetical protein | ND | ND | ND | ND |
| SO_4412 | 4606141 | conserved domain protein | ND | ND | ND | ND |
| SO_4413 | 4606608 | conserved hypothetical protein | ND | ND | ND | ND |
| SO_4414 | 4607749 | conserved domain protein | ND | ND | P | ND |
| SO_4415 | 4608945 | hypothetical protein | ND | ND | ND | ND |
| SO_4416 | 4609071 | hypothetical protein | ND | ND | ND | ND |
| SO_4417 | 4609208 | anaerobic C4-dicarboxylate membrane transporter | ND | ND | ND | ND |
| SO_4418 | 4610764 | trypanothione synthetase domain protein | ND | ND | ND | ND |
| SO_4419 | 4611433 | hypothetical protein | ND | ND | ND | ND |
| SO_4420 | 4612038 | peptidase, M23/M37 family | ND | ND | ND | ND |
| SO_4421 | 4612554 | conserved hypothetical protein | ND | ND | ND | ND |
| SO_4423 | 4615286 | conserved hypothetical protein | ND | ND | ND | ND |
| SO_4424 | 4615538 | hypothetical protein | ND | ND | ND | ND |
| SO_4425 | 4615800 | GGDEF family protein | ND | ND | ND | ND |
| SO_4427 | 4617831 | sensor histidine kinase | ND | ND | P | ND |
| SO_4428 | 4619137 | DNA-binding response regulator | P | ND | ND | ND |
| SO_4429 | 4619866 | hypothetical protein | ND | ND | ND | ND |
| SO_4430 | 4620880 | hypothetical protein | ND | ND | ND | ND |
| SO_4431 | 4621083 | conserved domain protein | ND | ND | ND | ND |
| SO_4432 | 4621273 | ISSod3, transposase | ND | ND | ND | ND |
| SO_4433 | 4622568 | conserved domain protein | ND | ND | ND | ND |
| SO_4434 | 4623379 | hypothetical protein | ND | ND | ND | ND |
| SO_4435 | 4624133 | hypothetical protein | ND | ND | ND | ND |
| SO_4436 | 4624326 | ISSod11, transposase | ND | ND | ND | ND |
| SO_4437 | 4625353 | ISSod3, transposase | ND | ND | ND | ND |
| SO_4438 | 4626642 | hypothetical protein | ND | ND | ND | ND |
| SO_4439 | 4627121 | conserved hypothetical protein | ND | ND | ND | ND |
| SO_4440 | 4629550 | hypothetical protein | ND | ND | ND | ND |
| SO_4441 | 4629990 | ISSod1, transposase OrfA | ND | ND | ND | ND |
| SO_4442 | 4630340 | ISSod1, transposase OrfB | ND | ND | ND | ND |
| SO_4443 | 4631172 | hypothetical protein | ND | ND | ND | ND |
| SO_4444 | 4632035 | capsular synthesis regulator component B, putative | ND | ND | ND | ND |
| SO_4445 | 4632770 | response regulator/sensor histidine kinase | ND | ND | ND | ND |
| SO_4446 | 4636205 | molybdenum ABC transporter, ATP-binding protein | ND | ND | ND | ND |
| SO_4447 | 4637289 | molybdenum ABC transporter, permease protein | ND | ND | ND | ND |
| SO_4448 | 4637978 | molybdenum ABC transporter, periplasmic molybdenum-binding protein | ND | ND | ND | ND |
| SO_4449 | 4638801 | molybdenum cofactor biosynthesis protein E | ND | ND | ND | ND |
| SO_4450 | 4639270 | molybdenum cofactor biosynthesis protein D | ND | ND | ND | ND |
| SO_4451 | 4639541 | molybdenum cofactor biosynthesis protein C | ND | ND | ND | ND |
| SO_4452 | 4640134 | molybdenum cofactor biosynthesis protein A | ND | ND | ND | ND |
| SO_4453 | 4641484 | electron transfer flavoprotein-ubiquinone oxidoreductase, putative | P | ND | P | ND |
| SO_4454 | 4643423 | methyl-accepting chemotaxis protein | ND | ND | ND | ND |
| SO_4455 | 4645427 | conserved hypothetical protein | ND | ND | ND | ND |
| SO_4456 | 4645820 | conserved hypothetical protein | ND | ND | ND | ND |
| SO_4457 | 4646588 | GGDEF domain protein | ND | ND | ND | ND |
| SO_4458 | 4648354 | carbohydrate kinase, PfkB family | ND | ND | ND | ND |
| SO_4459 | 4649379 | hypothetical protein | ND | ND | ND | ND |
| SO_4460 | 4649570 | hypothetical protein | ND | ND | ND | ND |
| SO_4461 | 4649831 | hypothetical protein | ND | ND | ND | ND |
| SO_4462 | 4650037 | hypothetical protein | ND | ND | ND | ND |
| SO_4463 | 4650574 | prolyl 4-hydroxylase, alpha subunit domain protein | ND | ND | ND | ND |
| SO_4464 | 4651709 | hypothetical protein | ND | ND | ND | ND |
| SO_4465 | 4651842 | conserved domain protein | ND | ND | ND | ND |
| SO_4466 | 4654062 | methyl-accepting chemotaxis protein | ND | ND | ND | ND |
| SO_4467 | 4656328 | conserved hypothetical protein | ND | ND | ND | ND |
| SO_4468 | 4656754 | transcriptional regulator, TetR family | ND | ND | ND | ND |
| SO_4469 | 4657483 | alcohol dehydrogenase, iron-containing | ND | ND | ND | ND |
| SO_4470 | 4658937 | conserved hypothetical protein | ND | ND | ND | ND |
| SO_4471 | 4659592 | nitrogen regulation protein | ND | ND | ND | ND |
| SO_4472 | 4660676 | nitrogen regulation protein NR(I) | ND | ND | ND | ND |
| SO_4473 | 4662161 | outer membrane protein, putative | ND | ND | ND | ND |
| SO_4474 | 4662881 | hypothetical protein | ND | ND | ND | ND |
| SO_4475 | 4663350 | cation efflux family protein | ND | ND | ND | ND |
| SO_4476 | 4664291 | spheroplast protein y precursor, putative | ND | ND | ND | ND |
| SO_4477 | 4665126 | transcriptional regulatory protein CpxR | ND | ND | ND | ND |
| SO_4478 | 4665827 | sensor protein CpxA | ND | ND | ND | ND |
| SO_4479 | 4667254 | sigma-54 dependent transcriptional regulator | ND | ND | ND | ND |
| SO_4480 | 4669472 | aldehyde dehydrogenase | ND | ND | ND | P |
| SO_4481 | 4671086 | hypothetical protein | ND | ND | ND | ND |
| SO_4482 | 4671618 | hypothetical protein | ND | ND | ND | ND |
| SO_4483 | 4672247 | cytochrome b, putative | ND | ND | ND | ND |
| SO_4484 | 4672877 | cytochrome c-type protein Shp | ND | ND | ND | ND |
| SO_4485 | 4673365 | diheme cytochrome c | ND | ND | ND | ND |
| SO_4486 | 4673921 | conserved hypothetical protein | ND | ND | ND | ND |
| SO_4487 | 4674187 | DNA-binding response regulator | ND | ND | ND | ND |
| SO_4488 | 4674852 | sensor histidine kinase | ND | ND | ND | ND |
| SO_4489 | 4676296 | acetyltransferase, GNAT family | ND | ND | ND | ND |
| SO_4490 | 4676855 | hypothetical protein | ND | ND | ND | ND |
| SO_4491 | 4677088 | hypothetical protein | ND | ND | ND | ND |
| SO_4492 | 4677431 | conserved hypothetical protein | ND | ND | P | ND |
| SO_4494 | 4678590 | hypothetical protein | ND | ND | ND | ND |
| SO_4495 | 4684562 | hypothetical protein | ND | ND | ND | ND |
| SO_4496 | 4684839 | hypothetical protein | ND | ND | ND | ND |
| SO_4497 | 4685644 | ISSod5, transposase | ND | ND | ND | ND |
| SO_4498 | 4686932 | hypothetical protein | ND | ND | ND | ND |
| SO_4499 | 4687326 | hypothetical protein | ND | ND | ND | ND |
| SO_4500 | 4693672 | hypothetical protein | ND | ND | ND | ND |
| SO_4501 | 4693949 | hypothetical protein | ND | ND | ND | ND |
| SO_4502 | 4694289 | conserved domain protein | ND | ND | ND | ND |
| SO_4503 | 4695252 | formate dehydrogenase accessory protein FdhD, putative | ND | ND | ND | ND |
| SO_4504 | 4696444 | conserved hypothetical protein | ND | ND | ND | ND |
| SO_4505 | 4696908 | conserved hypothetical protein | ND | ND | ND | ND |
| SO_4506 | 4697735 | iron-sulfur cluster-binding protein | ND | ND | P | ND |
| SO_4507 | 4699422 | TorA specific chaperone, putative | ND | ND | ND | ND |
| SO_4508 | 4700262 | hypothetical protein | ND | ND | ND | ND |
| SO_4509 | 4700477 | formate dehydrogenase, alpha subunit | ND | ND | ND | ND |
| SO_4510 | 4703353 | formate dehydrogenase, iron-sulfur subunit | ND | ND | ND | ND |
| SO_4511 | 4703999 | formate dehydrogenase, C subunit, putative | ND | ND | ND | ND |
| SO_4512 | 4705407 | conserved hypothetical protein | ND | ND | ND | ND |
| SO_4513 | 4705625 | formate dehydrogenase, alpha subunit | P | ND | P | P |
| SO_4514 | 4708507 | formate dehydrogenase, iron-sulfur subunit | ND | ND | ND | ND |
| SO_4515 | 4709153 | formate dehydrogenase, C subunit, putative | ND | ND | ND | ND |
| SO_4516 | 4710223 | ferric vibriobactin receptor | ND | ND | P | ND |
| SO_4517 | 4712325 | hypothetical protein | ND | ND | ND | ND |
| SO_4518 | 4712839 | hypothetical protein | ND | ND | ND | ND |
| SO_4519 | 4713116 | sodium-dependent transporter | ND | ND | ND | ND |
| SO_4520 | 4714407 | oxygen-independent coproporphyrinogen III oxidase, putative | P | ND | P | ND |
| SO_4521 | 4715808 | hypothetical protein | ND | ND | ND | ND |
| SO_4522 | 4716126 | conserved hypothetical protein | ND | ND | ND | ND |
| SO_4523 | 4717121 | iron-regulated outer membrane virulence protein | ND | ND | P | ND |
| SO_4524 | 4719235 | transcriptional regulator, LysR family | ND | ND | ND | ND |
| SO_4525 | 4720294 | conserved hypothetical protein | ND | ND | ND | ND |
| SO_4527 | 4722086 | integral membrane domain protein | ND | ND | ND | ND |
| SO_4528 | 4723037 | hypothetical protein | ND | ND | ND | ND |
| SO_4529 | 4724172 | RNA methyltransferase, TrmH family, group 2 | ND | ND | ND | ND |
| SO_4530 | 4724770 | hypothetical protein | ND | ND | ND | ND |
| SO_4531 | 4725800 | hypothetical protein | ND | ND | ND | ND |
| SO_4532 | 4726098 | ISSod10, transposase OrfB | ND | ND | ND | ND |
| SO_4533 | 4726645 | ISSod10, transposase OrfA | ND | ND | ND | ND |
| SO_4535 | 4727779 | hypothetical protein | ND | ND | ND | ND |
| SO_4536 | 4728233 | hypothetical protein | ND | ND | ND | ND |
| SO_4537 | 4728580 | peptidase, putative | ND | ND | P | ND |
| SO_4539 | 4734044 | serine protease, subtilase family | ND | ND | ND | ND |
| SO_4540 | 4738982 | hypothetical protein | ND | ND | ND | ND |
| SO_4541 | 4739108 | hypothetical protein | ND | ND | ND | ND |
| SO_4542 | 4739232 | transcriptional regulator, LysR family | ND | ND | ND | ND |
| SO_4543 | 4740350 | hypothetical protein | ND | ND | ND | ND |
| SO_4544 | 4741479 | hypothetical protein | ND | ND | ND | ND |
| SO_4545 | 4742010 | ISSod4, transposase | ND | ND | ND | ND |
| SO_4546 | 4743553 | hypothetical protein | ND | ND | ND | ND |
| SO_4547 | 4743909 | hypothetical protein | ND | ND | ND | ND |
| SO_4548 | 4745035 | transposase, IS110 family | ND | ND | ND | ND |
| SO_4549 | 4746394 | hypothetical protein | ND | ND | ND | ND |
| SO_4550 | 4746578 | methyltransferase domain protein | ND | ND | ND | ND |
| SO_4551 | 4747284 | conserved hypothetical protein | ND | ND | ND | ND |
| SO_4552 | 4747944 | sensory box protein | ND | ND | ND | ND |
| SO_4553 | 4750166 | hypothetical protein | ND | ND | ND | ND |
| SO_4554 | 4750361 | conserved hypothetical protein | ND | ND | ND | ND |
| SO_4555 | 4751075 | drug resistance transporter, Bcr/CflA family protein | ND | ND | ND | ND |
| SO_4556 | 4752399 | transcriptional regulator, LysR family | ND | ND | ND | ND |
| SO_4557 | 4753426 | methyl-accepting chemotaxis protein | P | ND | P | ND |
| SO_4558 | 4755742 | hypothetical protein | ND | ND | ND | ND |
| SO_4559 | 4756096 | conserved domain protein | ND | ND | ND | ND |
| SO_4560 | 4757586 | conserved hypothetical protein | ND | ND | ND | ND |
| SO_4561 | 4758485 | conserved hypothetical protein | ND | ND | P | ND |
| SO_4562 | 4759109 | conserved hypothetical protein | ND | ND | ND | ND |
| SO_4563 | 4759697 | conserved hypothetical protein | ND | ND | ND | ND |
| SO_4564 | 4760828 | TonB2 protein, putative | ND | ND | ND | ND |
| SO_4565 | 4761513 | transporter, putative | ND | ND | ND | ND |
| SO_4566 | 4762822 | hypothetical protein | ND | ND | ND | ND |
| SO_4567 | 4763020 | transcriptional regulator, AsnC family | ND | ND | ND | ND |
| SO_4568 | 4763717 | formate-dependent nitrite reductase, nrfD protein | ND | ND | ND | ND |
| SO_4570 | 4764722 | conserved domain protein | ND | ND | ND | ND |
| SO_4571 | 4765360 | transcriptional regulator, LysR family | ND | ND | ND | ND |
| SO_4572 | 4766497 | cytochrome c, putative | ND | ND | ND | ND |
| SO_4573 | 4766990 | 2-succinyl-6-hydroxy-2, 4-cyclohexadiene-1-carboxylic acid synthase/2-oxoglutarate decarboxylase | P | ND | ND | ND |
| SO_4574 | 4768711 | hydrolase, alpha/beta fold family | ND | ND | ND | ND |
| SO_4575 | 4769562 | O-succinylbenzoate-CoA synthase | ND | ND | ND | ND |
| SO_4576 | 4770745 | O-succinylbenzoic acid--CoA ligase, putative | ND | ND | ND | ND |
| SO_4577 | 4772183 | conserved domain protein | ND | ND | ND | ND |
| SO_4578 | 4772518 | transposase, putative | ND | ND | ND | ND |
| SO_4579 | 4773582 | fimbrial protein, putative | ND | ND | ND | ND |
| SO_4580 | 4774668 | ISSod1, transposase OrfB | ND | ND | ND | ND |
| SO_4581 | 4775537 | ISSod1, transposase OrfA | ND | ND | ND | ND |
| SO_4582 | 4776486 | ISSod4, transposase | ND | ND | ND | ND |
| SO_4583 | 4778232 | RNA polymerase sigma-32 factor | ND | ND | ND | ND |
| SO_4584 | 4779320 | cell division ABC transporter, permease protein FtsX | ND | ND | ND | ND |
| SO_4585 | 4780282 | cell division ABC transporter, ATP-binding protein FtsE | ND | ND | ND | ND |
| SO_4586 | 4780987 | cell division protein FtsY | ND | ND | ND | ND |
| SO_4587 | 4782862 | conserved hypothetical protein TIGR00095 | ND | ND | ND | ND |
| SO_4588 | 4783436 | conserved hypothetical protein | ND | ND | ND | ND |
| SO_4589 | 4783665 | transcriptional regulator, AraC family | ND | ND | ND | ND |
| SO_4590 | 4784767 | isochorismatase family protein | ND | ND | ND | ND |
| SO_4591 | 4785363 | tetraheme cytochrome c | ND | ND | ND | ND |
| SO_4592 | 4786336 | hypothetical protein | ND | ND | ND | ND |
| SO_4593 | 4786793 | hypothetical protein | ND | ND | ND | ND |
| SO_4594 | 4787299 | hypothetical protein | ND | ND | ND | ND |
| SO_4595 | 4787869 | hypothetical protein | ND | ND | ND | ND |
| SO_4596 | 4788013 | copper-transporting ATPase domain protein | ND | ND | ND | ND |
| SO_4597 | 4788361 | heavy metal efflux system protein, putative | ND | ND | ND | ND |
| SO_4598 | 4789789 | heavy metal efflux pump, CzcA family | ND | ND | ND | ND |
| SO_4599 | 4793172 | ribonuclease, T2 family | ND | ND | ND | ND |
| SO_4600 | 4794347 | antigen, putative | ND | ND | ND | ND |
| SO_4601 | 4795342 | tryptophan-specific transport protein | ND | ND | ND | ND |
| SO_4602 | 4796591 | glycerol-3-phosphate acyltransferase | ND | ND | ND | ND |
| SO_4603 | 4799182 | LexA repressor | ND | ND | ND | P |
| SO_4604 | 4799799 | conserved hypothetical protein | ND | ND | ND | ND |
| SO_4605 | 4800316 | hypothetical protein | ND | ND | ND | ND |
| SO_4606 | 4800716 | cytochrome c oxidase, subunit II | ND | ND | ND | ND |
| SO_4607 | 4802272 | cytochrome c oxidase, subunit I | ND | ND | ND | ND |
| SO_4608 | 4803866 | cytochrome c oxidase assembly protein coxG | ND | ND | ND | ND |
| SO_4609 | 4804444 | cytochrome c oxidase subunit III | ND | ND | ND | ND |
| SO_4610 | 4805366 | hypothetical protein | ND | ND | ND | ND |
| SO_4611 | 4805595 | conserved hypothetical protein | ND | ND | ND | ND |
| SO_4612 | 4806517 | conserved hypothetical protein | ND | ND | ND | ND |
| SO_4613 | 4807062 | cytochrome oxidase assembly protein, putative | ND | ND | ND | ND |
| SO_4614 | 4808057 | protoheme IX farnesyltransferase | ND | ND | ND | ND |
| SO_4615 | 4809010 | SCO1/SenC family protein | ND | ND | ND | ND |
| SO_4616 | 4809696 | polysaccharide deacetylase family protein | ND | ND | ND | ND |
| SO_4617 | 4810765 | DNA-damage-inducible protein F | ND | ND | ND | ND |
| SO_4618 | 4812210 | prolyl oligopeptidase family protein | ND | ND | ND | ND |
| SO_4619 | 4814210 | yhgI protein | ND | ND | ND | ND |
| SO_4620 | 4815040 | fumarate reductase, flavoprotein subunit precursor | ND | ND | ND | ND |
| SO_4621 | 4816881 | hypothetical protein | ND | ND | ND | ND |
| SO_4622 | 4817820 | sensor histidine kinase | ND | ND | ND | ND |
| SO_4623 | 4819048 | DNA-binding response regulator | ND | ND | ND | ND |
| SO_4624 | 4819728 | transcriptional regulator, LuxR family | ND | ND | ND | ND |
| SO_4625 | 4820646 | competence protein ComF | ND | ND | ND | ND |
| SO_4626 | 4821559 | bioH protein | ND | ND | ND | ND |
| SO_4627 | 4822313 | hypothetical protein | ND | ND | ND | ND |
| SO_4628 | 4822919 | sulfatase | ND | ND | ND | ND |
| SO_4629 | 4825116 | conserved hypothetical protein | P | ND | P | ND |
| SO_4630 | 4827495 | hypothetical protein | ND | ND | ND | ND |
| SO_4631 | 4827600 | transcription elongation factor GreB | ND | ND | ND | ND |
| SO_4632 | 4828203 | hypothetical protein | ND | ND | ND | ND |
| SO_4633 | 4828759 | transcriptional regulatory protein OmpR | ND | ND | ND | ND |
| SO_4634 | 4829525 | osmolarity sensor protein EnvZ | ND | ND | ND | ND |
| SO_4635 | 4830958 | methyl-accepting chemotaxis protein | ND | ND | ND | ND |
| SO_4636 | 4833017 | lipoprotein, putative | ND | ND | ND | ND |
| SO_4637 | 4834383 | DNA-binding response regulator | ND | ND | ND | ND |
| SO_4638 | 4835023 | sensor histidine kinase | ND | ND | ND | ND |
| SO_4639 | 4836406 | conserved hypothetical protein | ND | ND | ND | ND |
| SO_4640 | 4836897 | antioxidant, AhpC/Tsa family | ND | ND | P | ND |
| SO_4641 | 4837815 | conserved hypothetical protein | ND | ND | ND | ND |
| SO_4642 | 4838108 | conserved hypothetical protein | ND | ND | ND | ND |
| SO_4643 | 4838507 | hypothetical protein | ND | ND | ND | ND |
| SO_4644 | 4839084 | hypothetical protein | ND | ND | ND | ND |
| SO_4645 | 4841290 | hypothetical protein | ND | ND | ND | ND |
| SO_4646 | 4842575 | hypothetical protein | ND | ND | ND | ND |
| SO_4647 | 4842876 | DNA-binding response regulator | ND | ND | ND | ND |
| SO_4648 | 4843564 | sensor histidine kinase | ND | ND | ND | ND |
| SO_4649 | 4845079 | conserved hypothetical protein | ND | ND | ND | ND |
| SO_4650 | 4847044 | conserved hypothetical protein | ND | ND | ND | ND |
| SO_4651 | 4847760 | conserved hypothetical protein | ND | ND | ND | ND |
| SO_4652 | 4848018 | sulfate ABC transporter, periplasmic sulfate-binding protein | ND | ND | ND | ND |
| SO_4653 | 4849037 | sulfate ABC transporter, permease protein | ND | ND | ND | ND |
| SO_4654 | 4849923 | sulfate ABC transporter, permease protein | ND | ND | ND | ND |
| SO_4655 | 4850802 | sulfate ABC transporter, ATP-binding protein | ND | ND | ND | ND |
| SO_4656 | 4851926 | hypothetical protein | ND | ND | ND | ND |
| SO_4658 | 4857581 | conserved hypothetical protein | ND | ND | ND | ND |
| SO_4659 | 4858517 | conserved hypothetical protein | ND | ND | ND | ND |
| SO_4660 | 4859473 | transglycosylase SLT domain protein | ND | ND | ND | ND |
| SO_4661 | 4860192 | hypothetical protein | ND | ND | ND | ND |
| SO_4662 | 4861279 | lemA protein | ND | ND | ND | ND |
| SO_4663 | 4862067 | hypothetical protein | P | ND | ND | ND |
| SO_4664 | 4862312 | conserved hypothetical protein | ND | ND | ND | ND |
| SO_4665 | 4862607 | hypothetical protein | ND | ND | ND | ND |
| SO_4666 | 4863318 | cytochrome c | ND | ND | P | ND |
| SO_4667 | 4864111 | GTP-binding protein EngB | ND | ND | ND | ND |
| SO_4668 | 4865509 | hypothetical protein | ND | ND | ND | ND |
| SO_4669 | 4865820 | DNA polymerase I | P | P | P | P |
| SO_4670 | 4868974 | enhancing lycopene biosynthesis protein | ND | ND | ND | ND |
| SO_4671 | 4869710 | glpG protein | ND | ND | ND | ND |
| SO_4672 | 4870549 | glpE protein | ND | ND | ND | ND |
| SO_4673 | 4870953 | threonine 3-dehydrogenase | ND | ND | ND | ND |
| SO_4674 | 4871990 | 2-amino-3-ketobutyrate coenzyme A ligase | P | P | ND | P |
| SO_4675 | 4873469 | transcriptional regulator, TetR family | ND | ND | ND | ND |
| SO_4676 | 4874417 | 3-deoxy-D-manno-octulosonic-acid (KDO) transferase | ND | ND | ND | ND |
| SO_4677 | 4875707 | conserved hypothetical protein | ND | ND | ND | ND |
| SO_4678 | 4876548 | heptosyl transferase, glycosyltransferase family 9 protein | ND | ND | ND | ND |
| SO_4679 | 4877644 | glycosyl transferase, group 1 family protein | ND | ND | ND | ND |
| SO_4680 | 4878749 | conserved hypothetical protein | ND | ND | ND | ND |
| SO_4681 | 4879861 | glycosyl transferase, group 1 family protein | ND | ND | ND | ND |
| SO_4682 | 4881325 | glycosyl transferase, group 1 family protein | ND | ND | ND | ND |
| SO_4683 | 4882413 | hypothetical protein | ND | ND | ND | ND |
| SO_4684 | 4882671 | phosphopantetheine adenylyltransferase | ND | ND | ND | ND |
| SO_4685 | 4883228 | conserved hypothetical protein | ND | ND | ND | ND |
| SO_4686 | 4885039 | NAD dependent epimerase/dehydratase family protein | ND | ND | ND | ND |
| SO_4687 | 4886071 | UDP-glucose 6-dehydrogenase | ND | ND | ND | ND |
| SO_4688 | 4887469 | glycosyl transferase, group 2 family protein | ND | ND | ND | ND |
| SO_4689 | 4888202 | conserved hypothetical protein | ND | ND | ND | ND |
| SO_4690 | 4889105 | conserved hypothetical protein | ND | ND | ND | ND |
| SO_4691 | 4891195 | hypothetical protein | ND | ND | ND | ND |
| SO_4692 | 4891476 | AcrB/AcrD/AcrF family protein | ND | P | ND | ND |
| SO_4693 | 4894620 | multidrug resistance protein, AcrA/AcrE family | P | P | ND | ND |
| SO_4694 | 4896536 | hypothetical protein | ND | ND | ND | ND |
| SO_4695 | 4897257 | hypothetical protein | ND | ND | ND | ND |
| SO_4696 | 4897607 | conserved hypothetical protein | ND | ND | ND | ND |
| SO_4697 | 4898499 | glutathione S-transferase | ND | ND | ND | ND |
| SO_4698 | 4899159 | conserved hypothetical protein | ND | ND | ND | ND |
| SO_4699 | 4899826 | oligopeptidase A | P | P | ND | P |
