# Supplementary material for: An Empirical Strategy for Characterizing Bacterial Proteomes across Species in the Absence of Genomic Sequences
Source: PLoS One. 2010 Nov 12;5(11):e13968. doi: 10.1371/journal.pone.0013968 (PMC2980473; doi:10.1371/journal.pone.0013968)
Supplement: Table S3 — S. putrefaciens CN32 loci with poor proteome coverage from analysis with the Columbia River Shewanella isolates. ND indicates Not Detected, P indicates Present. (0.47 MB DOC) [file pone.0013968.s003.doc]

| Locus | Genome Start | Description | HRCR1 | HRCR2 | HRCR4 | HRCR5 |
| --- | --- | --- | --- | --- | --- | --- |
| CN32_0088 | 100033 | conserved hypothetical protein | ND | ND | ND | ND |
| CN32_0089 | 100589 | Glutathione S-transferase, N-terminal domain | ND | ND | ND | ND |
| CN32_0090 | 102007 | efflux transporter, RND family, MFP subunit | Present | Present | ND | ND |
| CN32_0091 | 103162 | transporter, hydrophobe/amphiphile efflux-1 (HAE1) family | ND | ND | Present | ND |
| CN32_0092 | 106425 | conserved hypothetical protein | ND | ND | ND | ND |
| CN32_0093 | 106745 | conserved hypothetical protein | ND | ND | ND | ND |
| CN32_0094 | 107146 | AbgT putative transporter | ND | ND | ND | ND |
| CN32_0095 | 109245 | LemA family protein | ND | ND | ND | ND |
| CN32_0096 | 109915 | Lytic transglycosylase, catalytic | ND | ND | ND | ND |
| CN32_0097 | 110661 | conserved hypothetical protein | ND | ND | ND | ND |
| CN32_0098 | 110989 | protein of unknown function DUF519 | ND | ND | ND | ND |
| CN32_0099 | 111971 | diguanylate cyclase/phosphodiesterase | ND | ND | ND | ND |
| CN32_0100 | 114456 | protein of unknown function DUF548 | ND | ND | ND | ND |
| CN32_0101 | 115816 | hypothetical protein | ND | ND | ND | ND |
| CN32_0102 | 116375 | hypothetical protein | ND | ND | ND | ND |
| CN32_0103 | 116931 | hypothetical protein | ND | ND | ND | ND |
| CN32_0104 | 117266 | acriflavin resistance protein | ND | ND | ND | ND |
| CN32_0105 | 120441 | efflux transporter, RND family, MFP subunit | ND | ND | ND | ND |
| CN32_0106 | 121532 | TonB-dependent siderophore receptor | ND | ND | ND | ND |
| CN32_0107 | 124135 | peptidase M48, Ste24p | ND | ND | ND | ND |
| CN32_0108 | 125794 | conserved hypothetical protein | ND | ND | ND | ND |
| CN32_0109 | 126451 | Glyoxalase/bleomycin resistance protein/dioxygenase | ND | ND | ND | ND |
| CN32_0110 | 126879 | conserved hypothetical protein | ND | ND | ND | ND |
| CN32_0111 | 127621 | transcriptional regulator, LysR family | ND | ND | ND | ND |
| CN32_0112 | 128733 | short-chain dehydrogenase/reductase SDR | ND | ND | ND | ND |
| CN32_0113 | 129778 | hypothetical protein | ND | ND | ND | ND |
| CN32_0114 | 130468 | cytochrome c biogenesis protein, transmembrane region | ND | ND | ND | ND |
| CN32_0115 | 132749 | DSBA oxidoreductase | ND | ND | ND | ND |
| CN32_0116 | 133599 | Redoxin domain protein | ND | ND | ND | ND |
| CN32_0117 | 134168 | protein of unknown function DUF1090 | ND | ND | ND | ND |
| CN32_0118 | 134709 | MscS Mechanosensitive ion channel | ND | ND | ND | ND |
| CN32_0119 | 135974 | Lysine exporter protein (LYSE/YGGA) | ND | ND | ND | ND |
| CN32_0120 | 136618 | phospholipid/glycerol acyltransferase | ND | ND | ND | ND |
| CN32_0121 | 137302 | conserved hypothetical protein | ND | ND | ND | ND |
| CN32_0122 | 137763 | protein of unknown function DUF833 | ND | ND | ND | ND |
| CN32_0123 | 138608 | conserved hypothetical protein | ND | ND | ND | ND |
| CN32_0124 | 140628 | multiple antibiotic resistance (MarC)-related protein | ND | ND | ND | ND |
| CN32_0125 | 141509 | peptidase M6, immune inhibitor A | ND | ND | ND | ND |
| CN32_0126 | 144195 | chorismate lyase | ND | ND | ND | ND |
| CN32_0127 | 144870 | flagellar basal body-associated protein FliL | ND | ND | ND | ND |
| CN32_0128 | 145349 | protein of unknown function DUF890 | ND | ND | ND | ND |
| CN32_0129 | 146543 | conserved hypothetical protein | ND | ND | ND | ND |
| CN32_0130 | 147273 | [molybdopterin synthase] sulfurylase | ND | ND | ND | ND |
| CN32_0131 | 148044 | molybdopterin molybdochelatase | ND | ND | ND | ND |
| CN32_0132 | 149639 | Ferritin, Dps family protein | ND | ND | ND | ND |
| CN32_0133 | 150238 | diguanylate cyclase/phosphodiesterase with PAS/PAC sensor(s) | ND | ND | ND | ND |
| CN32_0134 | 154012 | 3,4-dihydroxy-2-butanone 4-phosphate synthase | ND | ND | ND | ND |
| CN32_0135 | 155253 | Oligopeptidase B | ND | ND | ND | Present |
| CN32_0136 | 157399 | Excinuclease ABC, C subunit domain protein | ND | ND | ND | ND |
| CN32_0137 | 158259 | conserved hypothetical protein | ND | ND | ND | ND |
| CN32_0138 | 159624 | hypothetical protein | ND | ND | ND | ND |
| CN32_0139 | 160379 | conserved hypothetical protein | ND | ND | ND | ND |
| CN32_0140 | 160649 | conserved hypothetical protein | ND | ND | ND | ND |
| CN32_0141 | 161124 | lipoprotein, putative | ND | ND | ND | ND |
| CN32_0142 | 162172 | Methyltransferase type 12 | ND | ND | ND | ND |
| CN32_0143 | 162965 | peptidase M14, carboxypeptidase A | ND | ND | ND | ND |
| CN32_0144 | 165116 | conserved hypothetical protein | ND | ND | ND | ND |
| CN32_0145 | 166470 | sodium:dicarboxylate symporter | ND | ND | ND | ND |
| CN32_0146 | 168175 | hypothetical protein | ND | ND | ND | ND |
| CN32_0147 | 168674 | AbgT putative transporter | ND | ND | ND | ND |
| CN32_0148 | 170535 | hypothetical protein | ND | ND | ND | ND |
| CN32_0149 | 172225 | heavy metal efflux pump, CzcA family | ND | ND | ND | ND |
| CN32_0150 | 175384 | efflux transporter, RND family, MFP subunit | ND | ND | ND | ND |
| CN32_0151 | 176935 | copper-transporting ATPase domain protein | ND | ND | ND | ND |
| CN32_0152 | 177414 | conserved hypothetical protein | ND | ND | ND | ND |
| CN32_0153 | 177983 | site-specific recombinase, phage integrase family | ND | ND | ND | ND |
| CN32_0154 | 178519 | site-specific DNA-methyltransferase, type I modification | ND | ND | ND | ND |
| CN32_0155 | 179220 | AAA ATPase | ND | ND | ND | ND |
| CN32_0156 | 180847 | protein of unknown function DUF45 | ND | ND | ND | ND |
| CN32_0157 | 181665 | type I site-specific deoxyribonuclease, HsdR family | ND | ND | ND | ND |
| CN32_0158 | 184966 | KAP P-loop domain protein | ND | ND | ND | ND |
| CN32_0159 | 186313 | putative deoxyguanosinetriphosphate triphosphohydrolase | ND | ND | ND | ND |
| CN32_0160 | 187701 | restriction modification system DNA specificity domain | ND | ND | ND | ND |
| CN32_0161 | 188890 | type I restriction-modification system, M subunit | ND | ND | ND | ND |
| CN32_0162 | 190451 | conserved hypothetical protein | ND | ND | ND | ND |
| CN32_0163 | 191300 | hypothetical protein | ND | ND | ND | ND |
| CN32_0164 | 192103 | hypothetical protein | ND | ND | ND | ND |
| CN32_0165 | 192454 | AAA ATPase | ND | ND | ND | ND |
| CN32_0166 | 193415 | putative integrase protein | ND | ND | ND | ND |
| CN32_0167 | 195081 | Resolvase, N-terminal domain | ND | ND | ND | ND |
| CN32_0168 | 195909 | mercuric reductase | ND | ND | ND | ND |
| CN32_0169 | 197674 | mercuric transport protein periplasmic component | ND | ND | ND | ND |
| CN32_0170 | 198002 | Mercuric transport protein MerT | ND | ND | ND | ND |
| CN32_0171 | 198429 | putative transcriptional regulator, MerR family | ND | ND | ND | ND |
| CN32_0172 | 199130 | UMUC domain protein DNA-repair protein | ND | ND | ND | ND |
| CN32_0173 | 199833 | Integrase, catalytic region | ND | ND | ND | ND |
| CN32_0174 | 200699 | transposase IS3/IS911 family protein | ND | ND | ND | ND |
| CN32_0175 | 201013 | putative prophage repressor | ND | ND | ND | ND |
| CN32_0176 | 201597 | hypothetical protein | ND | ND | ND | ND |
| CN32_0177 | 201946 | prevent-host-death family protein | ND | ND | ND | ND |
| CN32_0178 | 202826 | phage integrase family protein | ND | ND | ND | ND |
| CN32_0179 | 203703 | conserved hypothetical protein | ND | ND | ND | ND |
| CN32_0180 | 204634 | hypothetical protein | ND | ND | ND | ND |
| CN32_0181 | 205738 | conserved hypothetical protein | ND | ND | ND | ND |
| CN32_0182 | 206070 | putative transcriptional regulator | ND | ND | ND | ND |
| CN32_0183 | 206732 | hypothetical protein | ND | ND | ND | ND |
| CN32_0184 | 207047 | hypothetical protein | ND | ND | ND | ND |
| CN32_0185 | 207856 | conserved hypothetical protein | ND | ND | ND | ND |
| CN32_0186 | 208506 | sulphate transporter | ND | ND | ND | ND |
| CN32_0187 | 210031 | UspA domain protein | ND | ND | ND | ND |
| CN32_0188 | 210976 | transcriptional regulator, TraR/DksA family | ND | ND | ND | ND |
| CN32_0189 | 211426 | Resolvase, N-terminal domain | ND | ND | ND | ND |
| CN32_0190 | 212216 | prevent-host-death family protein | ND | ND | ND | ND |
| CN32_0191 | 212476 | plasmid stabilization system | ND | ND | ND | ND |
| CN32_0192 | 212811 | transposase Tn3 family protein | ND | ND | ND | ND |
| CN32_0193 | 215929 | cointegrate resolution protein T | ND | ND | ND | ND |
| CN32_0194 | 216839 | putative transcriptional regulator, MerR family | ND | ND | ND | ND |
| CN32_0195 | 217342 | cation efflux system permease, putative | ND | ND | ND | ND |
| CN32_0196 | 218242 | lipoprotein signal peptidase | ND | ND | ND | ND |
| CN32_0197 | 218776 | transposase, IS204/IS1001/IS1096/IS1165 family protein | ND | ND | ND | ND |
| CN32_0198 | 220338 | iron-containing alcohol dehydrogenase | ND | ND | ND | ND |
| CN32_0199 | 221466 | aldehyde dehydrogenase | ND | ND | ND | ND |
| CN32_0200 | 222857 | Uncharacterized protein possibly involved in utilization of glycolate and propanediol-like protein | ND | ND | ND | ND |
| CN32_0201 | 223399 | Ethanolamine utilization protein EutN/carboxysome structural protein Ccml | ND | ND | ND | ND |
| CN32_0202 | 223682 | propanediol utilization | ND | ND | ND | ND |
| CN32_0203 | 224175 | Propanediol utilization protein | ND | ND | ND | ND |
| CN32_0204 | 224815 | microcompartments protein | ND | ND | ND | ND |
| CN32_0205 | 225709 | microcompartments protein | ND | ND | ND | ND |
| CN32_0206 | 226071 | MIP family channel protein | ND | ND | ND | ND |
| CN32_0207 | 226933 | glycyl-radical enzyme activating protein family | ND | ND | ND | ND |
| CN32_0208 | 227992 | pyruvate formate-lyase | ND | ND | ND | ND |
| CN32_0209 | 230560 | microcompartments protein | ND | ND | ND | ND |
| CN32_0210 | 231372 | microcompartments protein | ND | ND | ND | ND |
| CN32_0211 | 232687 | PTS system, mannose/fructose/sorbose family, IID subunit | ND | ND | ND | ND |
| CN32_0212 | 233558 | PTS system, mannose/fructose/sorbose family, IIC subunit | ND | ND | ND | ND |
| CN32_0213 | 234371 | PTS system sorbose subfamily IIB component | ND | ND | ND | ND |
| CN32_0214 | 234751 | transposase, IS4 family | ND | ND | ND | ND |
| CN32_0215 | 235737 | IS1 transposase | ND | ND | ND | ND |
| CN32_0216 | 235997 | Insertion element protein | ND | ND | ND | ND |
| CN32_0217 | 236424 | phage integrase family protein | ND | ND | ND | ND |
| CN32_0218 | 237269 | IS630 ORF | ND | ND | ND | ND |
| CN32_0219 | 238827 | transcriptional regulator, AraC family | ND | ND | ND | ND |
| CN32_0220 | 239957 | transcriptional regulator, MerR family | ND | ND | ND | ND |
| CN32_0221 | 240460 | cation efflux system permease, putative | ND | ND | ND | ND |
| CN32_0222 | 241360 | lipoprotein signal peptidase | ND | ND | ND | ND |
| CN32_0223 | 241894 | transposase, IS204/IS1001/IS1096/IS1165 family protein | ND | ND | ND | ND |
| CN32_0224 | 243253 | conserved hypothetical protein | ND | ND | ND | ND |
| CN32_0225 | 243729 | heavy metal efflux pump, CzcA family | ND | ND | ND | ND |
| CN32_0226 | 246861 | biotin/lipoyl attachment domain-containing protein | ND | ND | ND | ND |
| CN32_0227 | 247986 | outer membrane efflux protein | ND | ND | ND | ND |
| CN32_0228 | 249282 | hypothetical protein | ND | ND | ND | ND |
| CN32_0229 | 249734 | cation efflux system permease, putative | ND | ND | ND | ND |
| CN32_0230 | 250662 | putative transcriptional regulator, MerR family | ND | ND | ND | ND |
| CN32_0231 | 251158 | cation diffusion facilitator family transporter | ND | ND | ND | ND |
| CN32_0232 | 252041 | protein of unknown function UPF0060 | ND | ND | ND | ND |
| CN32_0233 | 252538 | hypothetical protein | ND | ND | ND | ND |
| CN32_0234 | 252957 | hypothetical protein | ND | ND | ND | ND |
| CN32_0235 | 253541 | conserved hypothetical protein | ND | ND | ND | ND |
| CN32_0236 | 254455 | hypothetical protein | ND | ND | ND | ND |
| CN32_0237 | 254905 | phage integrase family protein | ND | ND | ND | ND |
| CN32_0238 | 255998 | conserved hypothetical protein | ND | ND | ND | ND |
| CN32_0239 | 256659 | hypothetical protein | ND | ND | ND | ND |
| CN32_0240 | 257841 | hypothetical protein | ND | ND | ND | ND |
| CN32_0241 | 258257 | hypothetical protein | ND | ND | ND | ND |
| CN32_0242 | 258732 | hypothetical protein | ND | ND | ND | ND |
| CN32_0243 | 261081 | ISSod10, transposase OrfB | ND | ND | ND | ND |
| CN32_0244 | 261628 | ISSod10, transposase OrfA | ND | ND | ND | ND |
| CN32_0246 | 262745 | transposase, IS4 family | ND | ND | ND | ND |
| CN32_0247 | 264071 | conserved hypothetical protein | ND | ND | ND | ND |
| CN32_0248 | 264630 | protein of unknown function DUF411 | ND | ND | ND | ND |
| CN32_0249 | 265142 | copper-resistance protein, CopA family | ND | ND | ND | ND |
| CN32_0250 | 267172 | copper resistance B precursor | ND | ND | ND | ND |
| CN32_0251 | 268001 | copper resistance protein CopC | ND | ND | ND | ND |
| CN32_0252 | 268438 | copper resistance D domain protein | ND | ND | ND | ND |
| CN32_0253 | 269410 | putative orphan protein | ND | ND | ND | ND |
| CN32_0254 | 270036 | hypothetical protein | ND | ND | ND | ND |
| CN32_0255 | 271015 | outer membrane porin | ND | ND | ND | ND |
| CN32_0256 | 272702 | hypothetical protein | ND | ND | ND | ND |
| CN32_0257 | 273165 | multi-sensor signal transduction histidine kinase | ND | ND | ND | ND |
| CN32_0258 | 274633 | two component heavy metal response transcriptional regulator, winged helix family | ND | ND | ND | ND |
| CN32_0259 | 275503 | RND efflux system, outer membrane lipoprotein, NodT family | ND | ND | ND | ND |
| CN32_0260 | 276917 | periplasmic copper-binding protein | ND | ND | ND | ND |
| CN32_0261 | 277384 | efflux transporter, RND family, MFP subunit | ND | ND | ND | ND |
| CN32_0262 | 278687 | heavy metal efflux pump, CzcA family | ND | ND | ND | ND |
| CN32_0263 | 281929 | protein of unknown function DUF411 | ND | ND | ND | ND |
| CN32_0264 | 282487 | heavy metal translocating P-type ATPase | ND | ND | ND | ND |
| CN32_0265 | 284975 | conserved hypothetical protein | ND | ND | ND | ND |
| CN32_0266 | 285206 | peptidase M23B | ND | ND | ND | ND |
| CN32_0267 | 286232 | hypothetical protein | ND | ND | ND | ND |
| CN32_0269 | 287569 | likely secreted protein containing plastocyanin domain | ND | ND | ND | ND |
| CN32_0270 | 287930 | copper-translocating P-type ATPase | ND | ND | ND | ND |
| CN32_0271 | 290176 | putative transcriptional regulator, MerR family | ND | ND | ND | ND |
| CN32_0272 | 290767 | conserved hypothetical protein | ND | ND | ND | ND |
| CN32_0273 | 291293 | conserved hypothetical protein | ND | ND | ND | ND |
| CN32_0275 | 292164 | RNA-directed DNA polymerase (Reverse transcriptase) | ND | ND | ND | ND |
| CN32_0276 | 293225 | Integrase, catalytic region | ND | ND | ND | ND |
| CN32_0277 | 294097 | transposase IS3/IS911 family protein | ND | ND | ND | ND |
| CN32_0278 | 294467 | conserved hypothetical protein | ND | ND | ND | ND |
| CN32_0279 | 295147 | conserved hypothetical protein | ND | ND | ND | ND |
| CN32_0280 | 295331 | site-specific recombinase, phage integrase family | ND | ND | ND | ND |
| CN32_0281 | 295616 | conserved hypothetical protein | ND | ND | ND | ND |
| CN32_0282 | 297048 | heavy metal efflux pump, CzcA family | ND | ND | ND | ND |
| CN32_0283 | 300207 | efflux transporter, RND family, MFP subunit | ND | ND | ND | ND |
| CN32_0284 | 301767 | conserved hypothetical protein | ND | ND | ND | ND |
| CN32_0285 | 302279 | conserved hypothetical protein | ND | ND | ND | ND |
| CN32_0286 | 303207 | NapC/NirT cytochrome c domain protein | ND | ND | ND | ND |
| CN32_0287 | 303844 | protein of unknown function DUF1145 | ND | ND | ND | ND |
| CN32_0288 | 304103 | putative methyltransferase | ND | ND | ND | ND |
| CN32_0289 | 304982 | signal recognition particle-docking protein FtsY | ND | ND | ND | ND |
| CN32_0290 | 306434 | cell division ATP-binding protein FtsE | ND | ND | ND | ND |
| CN32_0291 | 307147 | cell division protein FtsX | ND | ND | ND | ND |
| CN32_0292 | 308360 | RNA polymerase, sigma 32 subunit, RpoH | ND | ND | ND | ND |
| CN32_0293 | 310518 | Fimbrial protein | ND | ND | ND | ND |
| CN32_0295 | 311981 | conserved hypothetical protein | ND | ND | ND | ND |
| CN32_0296 | 313743 | hypothetical protein | ND | ND | ND | ND |
| CN32_0297 | 314187 | fimbrial subunit | ND | ND | ND | ND |
| CN32_0298 | 315969 | Fimbrial protein | ND | ND | ND | ND |
| CN32_0299 | 316590 | pili assembly chaperone | ND | ND | ND | ND |
| CN32_0300 | 317329 | fimbrial biogenesis outer membrane usher protein | ND | ND | ND | ND |
| CN32_0301 | 319895 | fimbrial subunit | ND | ND | ND | ND |
| CN32_0302 | 321427 | pili assembly chaperone | ND | ND | ND | ND |
| CN32_0303 | 322756 | multi-sensor hybrid histidine kinase | ND | ND | ND | ND |
| CN32_0304 | 326343 | two component transcriptional regulator, LuxR family | ND | ND | ND | ND |
| CN32_0305 | 327334 | response regulator receiver modulated diguanylate phosphodiesterase | ND | ND | ND | ND |
| CN32_0306 | 328682 | conserved hypothetical protein | ND | ND | ND | ND |
| CN32_0307 | 329452 | O-succinylbenzoate-CoA ligase | ND | ND | ND | ND |
| CN32_0308 | 330872 | O-succinylbenzoate-CoA synthase | ND | ND | ND | ND |
| CN32_0309 | 331994 | alpha/beta hydrolase fold | ND | ND | ND | ND |
| CN32_0310 | 332782 | 2-succinyl-6-hydroxy-2, 4-cyclohexadiene-1-carboxylate synthase | ND | ND | ND | ND |
| CN32_0311 | 334686 | transcriptional regulator, LysR family | ND | ND | ND | ND |
| CN32_0312 | 336066 | conserved hypothetical protein | ND | ND | ND | ND |
| CN32_0313 | 336620 | formate-dependent nitrite reductase | ND | ND | ND | ND |
| CN32_0314 | 337089 | 4Fe-4S ferredoxin, iron-sulfur binding domain protein | ND | ND | ND | ND |
| CN32_0315 | 337772 | Polysulphide reductase, NrfD | ND | ND | ND | ND |
| CN32_0316 | 338992 | transcriptional regulator, AsnC family | ND | ND | ND | ND |
| CN32_0317 | 339673 | amino acid permease-associated region | ND | ND | ND | ND |
| CN32_0318 | 340918 | protein of unknown function DUF1332 | ND | ND | ND | ND |
| CN32_0319 | 341474 | conserved hypothetical protein | ND | ND | ND | ND |
| CN32_0320 | 342194 | conserved hypothetical protein | ND | ND | ND | ND |
| CN32_0321 | 342936 | Sel1 domain protein repeat-containing protein | ND | ND | ND | ND |
| CN32_0322 | 344400 | conserved hypothetical protein | ND | ND | ND | ND |
| CN32_0323 | 345092 | methyl-accepting chemotaxis sensory transducer | ND | ND | ND | ND |
| CN32_0324 | 347066 | transcriptional regulator, LysR family | ND | ND | ND | ND |
| CN32_0325 | 348151 | drug resistance transporter, Bcr/CflA subfamily | ND | ND | ND | ND |
| CN32_0326 | 349483 | DTW domain containing protein | ND | ND | ND | ND |
| CN32_0327 | 350449 | diguanylate cyclase/phosphodiesterase with PAS/PAC sensor(s) | ND | ND | ND | ND |
| CN32_0328 | 352876 | conserved hypothetical protein | ND | ND | ND | ND |
| CN32_0329 | 353300 | Methyltransferase type 11 | ND | ND | ND | ND |
| CN32_0330 | 354269 | TonB-dependent receptor | ND | ND | ND | ND |
| CN32_0331 | 356420 | formate dehydrogenase gamma subunit | ND | ND | ND | ND |
| CN32_0332 | 357483 | 4Fe-4S ferredoxin, iron-sulfur binding domain protein | ND | ND | ND | ND |
| CN32_0333 | 358082 | formate dehydrogenase alpha subunit | ND | ND | ND | ND |
| CN32_0334 | 360952 | twin-arginine translocation pathway signal | ND | ND | ND | ND |
| CN32_0335 | 361537 | formate dehydrogenase gamma subunit | ND | ND | ND | ND |
| CN32_0336 | 362615 | 4Fe-4S ferredoxin, iron-sulfur binding domain protein | ND | ND | ND | ND |
| CN32_0337 | 363238 | formate dehydrogenase alpha subunit | ND | ND | ND | ND |
| CN32_0338 | 366106 | conserved hypothetical protein | ND | ND | ND | ND |
| CN32_0339 | 366463 | cytoplasmic chaperone TorD family protein | ND | ND | ND | ND |
| CN32_0340 | 367151 | 4Fe-4S ferredoxin, iron-sulfur binding domain protein | ND | ND | ND | ND |
| CN32_0341 | 369000 | conserved hypothetical protein | ND | ND | ND | ND |
| CN32_0342 | 369656 | conserved hypothetical protein | ND | ND | ND | ND |
| CN32_0343 | 370426 | formate dehydrogenase, subunit FdhD | ND | ND | ND | ND |
| CN32_0344 | 371385 | DNA binding domain, excisionase family | ND | ND | ND | ND |
| CN32_0345 | 378641 | conserved hypothetical protein | ND | ND | ND | Present |
| CN32_0346 | 379564 | conserved hypothetical protein | ND | ND | ND | ND |
| CN32_0347 | 380239 | anion transporter | ND | ND | ND | ND |
| CN32_0348 | 381710 | fumarase | ND | ND | ND | ND |
| CN32_0349 | 383302 | response regulator receiver | ND | ND | ND | ND |
| CN32_0350 | 383969 | signal transduction histidine kinase regulating citrate/malate metabolism | ND | ND | ND | ND |
| CN32_0351 | 385820 | succinate dehydrogenase subunit D | ND | ND | ND | ND |
| CN32_0352 | 386198 | fumarate reductase, subunit C | ND | ND | ND | ND |
| CN32_0353 | 386583 | succinate dehydrogenase subunit B | ND | ND | ND | ND |
| CN32_0354 | 387323 | succinate dehydrogenase subunit A | ND | ND | ND | ND |
| CN32_0355 | 389401 | GCN5-related N-acetyltransferase | ND | ND | ND | ND |
| CN32_0356 | 389991 | integral membrane sensor signal transduction histidine kinase | ND | ND | ND | ND |
| CN32_0357 | 391312 | two component transcriptional regulator, winged helix family | ND | ND | ND | ND |
| CN32_0358 | 391971 | Propeptide, PepSY amd peptidase M4 | ND | ND | ND | ND |
| CN32_0359 | 392230 | diheme cytochrome c | ND | ND | ND | ND |
| CN32_0360 | 392884 | cytochrome c-type protein Shp | ND | ND | ND | ND |
| CN32_0361 | 393384 | cytochrome B561 | ND | ND | ND | ND |
| CN32_0362 | 394188 | conserved hypothetical protein | ND | ND | ND | ND |
| CN32_0363 | 394733 | conserved hypothetical protein | ND | ND | ND | ND |
| CN32_0364 | 395402 | Aldehyde dehydrogenase (NAD(+)) | ND | ND | ND | ND |
| CN32_0365 | 397183 | GAF modulated sigma54 specific transcriptional regulator, Fis family | ND | ND | ND | ND |
| CN32_0366 | 399127 | integral membrane sensor signal transduction histidine kinase | ND | ND | ND | ND |
| CN32_0367 | 400491 | two component transcriptional regulator, winged helix family | ND | ND | ND | ND |
| CN32_0368 | 401444 | protein of unknown function, Spy-related | ND | ND | ND | ND |
| CN32_0369 | 401986 | cation diffusion facilitator family transporter | ND | ND | ND | ND |
| CN32_0370 | 402886 | conserved hypothetical protein | ND | ND | ND | ND |
| CN32_0371 | 403434 | nitrogen metabolism transcriptional regulator, NtrC, Fis family | ND | ND | ND | ND |
| CN32_0372 | 404884 | signal transduction histidine kinase, nitrogen specific, NtrB | ND | ND | ND | ND |
| CN32_0373 | 406052 | conserved hypothetical protein | ND | ND | ND | ND |
| CN32_0374 | 406777 | glutathione S-transferase-like protein | ND | ND | ND | ND |
| CN32_0375 | 407300 | Glutathione S-transferase, N-terminal domain | ND | ND | ND | ND |
| CN32_0376 | 407799 | ThiJ/PfpI domain protein | ND | ND | ND | ND |
| CN32_0377 | 408563 | iron-containing alcohol dehydrogenase | ND | ND | ND | ND |
| CN32_0378 | 409863 | transcriptional regulator, TetR family | ND | ND | ND | ND |
| CN32_0379 | 410754 | protein of unknown function DUF1255 | ND | ND | ND | ND |
| CN32_0380 | 411424 | methyl-accepting chemotaxis sensory transducer | ND | ND | ND | ND |
| CN32_0381 | 413512 | protein of unknown function DUF323 | ND | ND | ND | ND |
| CN32_0382 | 415995 | conserved hypothetical protein | ND | ND | ND | ND |
| CN32_0383 | 416767 | PfkB domain protein | ND | ND | ND | ND |
| CN32_0384 | 417926 | diguanylate cyclase | ND | ND | ND | ND |
| CN32_0385 | 419515 | MOSC domain containing protein | ND | ND | ND | ND |
| CN32_0386 | 420247 | conserved hypothetical protein | ND | ND | ND | ND |
| CN32_0387 | 420671 | methyl-accepting chemotaxis sensory transducer | ND | ND | ND | ND |
