# Supplementary figures and images for: An Empirical Strategy for Characterizing Bacterial Proteomes across Species in the Absence of Genomic Sequences
Source: PLoS One. 2010 Nov 12;5(11):e13968. doi: 10.1371/journal.pone.0013968 (PMC2980473; doi:10.1371/journal.pone.0013968)

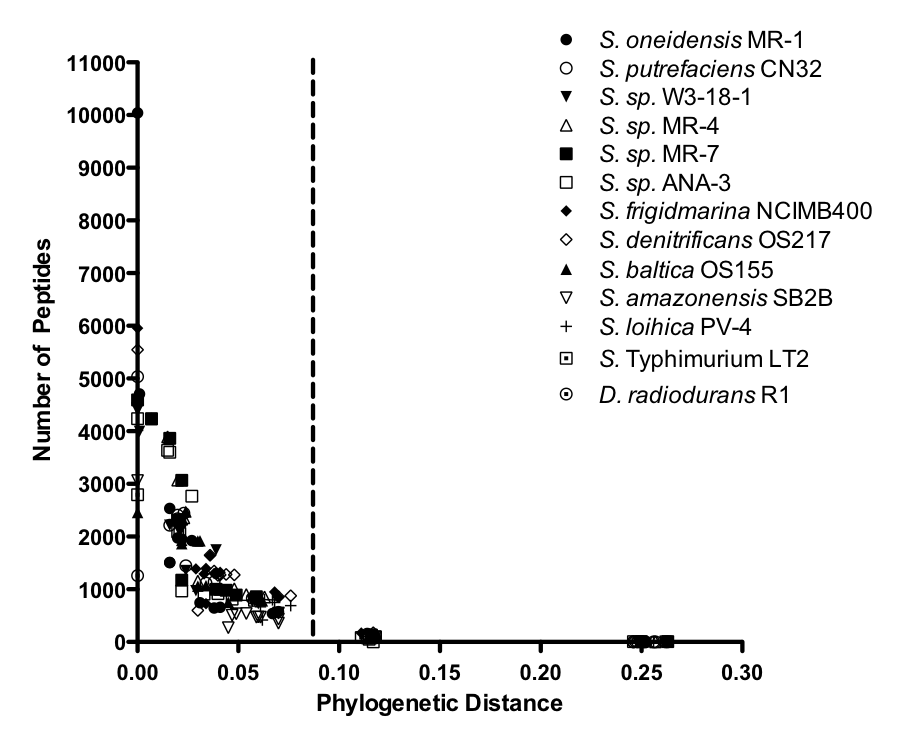

Supplement: Figure S1 — Plot of the number of peptide observations prior to normalization versus neighbor organism evolutionary distance. (0.11 MB TIF) [file pone.0013968.s004.tif]

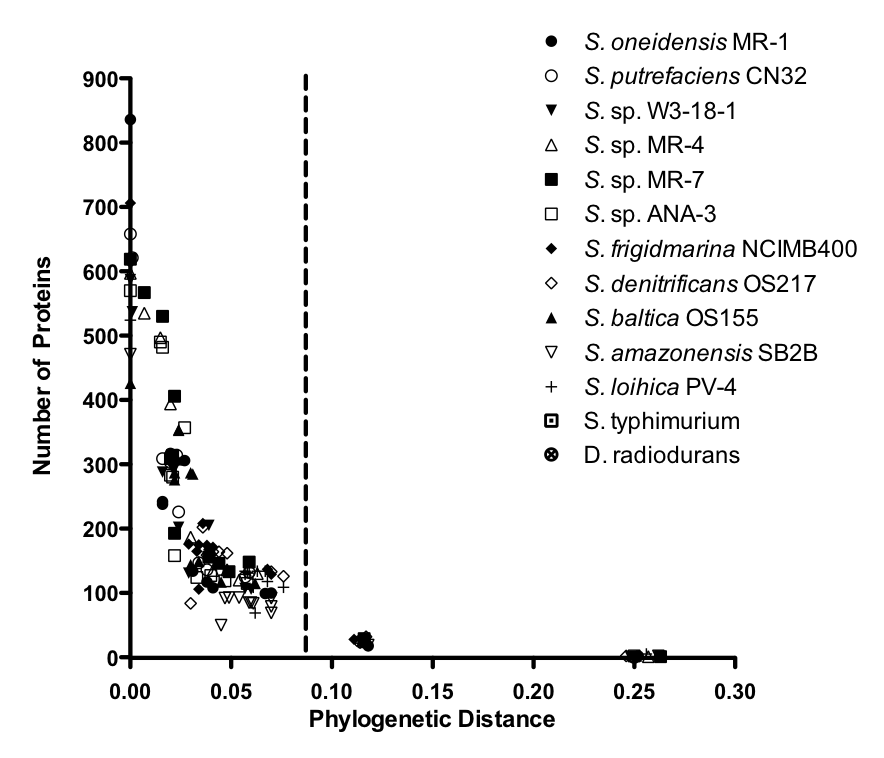

Supplement: Figure S2 — Plot of the number of protein observations prior to normalization versus neighbor organism evolutionary distance. (0.10 MB TIF) [file pone.0013968.s005.tif]
